# Single-cell RNA-seq landscape midbrain cell responses to red spotted grouper nervous necrosis virus infection

**DOI:** 10.1371/journal.ppat.1009665

**Published:** 2021-06-29

**Authors:** Qing Wang, Cheng Peng, Min Yang, Fengqi Huang, Xuzhuo Duan, Shaowen Wang, Huitao Cheng, Huirong Yang, Huihong Zhao, Qiwei Qin

**Affiliations:** 1 Joint Laboratory of Guangdong Province and Hong Kong Region on Marine Bioresource Conservation and Exploitation, College of Marine Sciences, South China Agricultural University, Guangzhou, China; 2 Guangdong Laboratory for Lingnan Modern Agriculture, Guangzhou, China; 3 Guangdong Key Laboratory of Animal Conservation and Resource Utilization, Guangdong Public Laboratory of Wild Animal Conservation and Utilization, Institute of Zoology, Academy of Sciences, Guangzhou, China; 4 Laboratory for Marine Biology and Biotechnology, Qingdao National Laboratory for Marine Science and Technology, Qingdao, China; National Institutes of Health, UNITED STATES

## Abstract

Viral nervous necrosis (VNN) is an acute and serious fish disease caused by nervous necrosis virus (NNV) which has been reported massive mortality in more than fifty teleost species worldwide. VNN causes damage of necrosis and vacuolation to central nervous system (CNS) cells in fish. It is difficult to identify the specific type of cell targeted by NNV, and to decipher the host immune response because of the functional diversity and highly complex anatomical and cellular composition of the CNS. In this study, we found that the red spotted grouper NNV (RGNNV) mainly attacked the midbrain of orange-spotted grouper (*Epinephelus coioides*). We conducted single-cell RNA-seq analysis of the midbrain of healthy and RGNNV-infected fish and identified 35 transcriptionally distinct cell subtypes, including 28 neuronal and 7 non-neuronal cell types. An evaluation of the subpopulations of immune cells revealed that macrophages were enriched in RGNNV-infected fish, and the transcriptional profiles of macrophages indicated an acute cytokine and inflammatory response. Unsupervised pseudotime analysis of immune cells showed that microglia transformed into M1-type activated macrophages to produce cytokines to reduce the damage to nerve tissue caused by the virus. We also found that RGNNV targeted neuronal cell types was GLU1 and GLU3, and we found that the key genes and pathways by which causes cell cytoplasmic vacuoles and autophagy significant enrichment, this may be the major route viruses cause cell death. These data provided a comprehensive transcriptional perspective of the grouper midbrain and the basis for further research on how viruses infect the teleost CNS.

## Author summary

NNV is one of the most contagious and economically devastating viruses of marine fish, which lead to severe central nervous system (CNS) damage. Because of the functional diversity and highly complex anatomical and cellular composition of the CNS, it is hard to identify the specific type of cell targeted by NNV, and to decipher the host immune response. In this study, we conducted single-cell RNA-seq analysis of the midbrain of healthy and RGNNV-infected fish and identified 35 transcriptionally distinct cell subtypes, including 28 neuronal and seven non-neuronal cell types. An evaluation of the subpopulations of immune cells revealed that macrophages were enriched in RGNNV-infected fish, and the transcriptional profiles of macrophages indicated an acute cytokine and inflammatory response. Unsupervised pseudotime analysis of immune cells showed that microglia transformed into M1-type activated macrophages to produce cytokines to reduce the damage to nerve tissue caused by the virus. Finally, we found that RGNNV targeted GLU1 and GLU3 neuronal cells, and we screened for genes that cause GLU1 and GLU3 cell vacuole morphology changes and death. These data provided a comprehensive transcriptional perspective of the grouper midbrain and the basis for further research on how viruses infect the teleost CNS.

## Introduction

Nervous necrosis viruses (NNVs) are non-enveloped positive-strand RNA viruses classified into the family *Nodaviridae* [1,2]. NNVs are about 25–30 nm in diameter, icosahedral, non-enveloped, and have a bipartite positive-sense RNA genome. The genome of NNV is bipartite, consisting of two positive-sense RNA molecules (RNA1 and RNA2). RNA1 encodes the RNA-dependent RNA polymerase (RdRp), which is responsible for viral genome replication, and RNA2 encodes the capsid protein (CP), which is the sole structural protein of NNV [3]. Fish infected with this virus show clinical signs that include abnormal swimming behavior and darkening of the fish [4]. NNVs can cause massive mortality of the larval and juvenile populations of more than 50 marine and freshwater teleost species [5–9], which illustrates their strong infectivity of a wide range of hosts.

Many viruses cause serious damage to the nervous system, including Japanese encephalitis virus [10], pseudorabies virus [11], Zika virus [12], herpes simplex virus (HSV) [13], porcine hemagglutinating encephalomyelitis virus (PHEV) [14], and these neuronal viral infections cause high mortality. Similarly, NNV infection can lead to severe central nervous system (CNS) damage. Vacuolization of the brain can occur in infected fish and is the main cause of their death [15,16]. Viral infection is a dynamic process driven by the interplay of antiviral cellular pathways and viral mechanisms, which have evolved to suppress antiviral activity. The membrane receptors and process of virus invasion are already well understood for many non-nervous system viruses, but little is known about the entry process and molecular signatures of CNS viruses.

The cellular heat shock cognate protein 70 (HSP70) [17] and cell surface sialic acid [18] are essential for NNV infection, and endosomal acidification also is required for effective infection [19]. However, these results do not provide information about the unique molecular signatures that arise when NNVs infect CNS cells. It is difficult to identify the specific type of cell targeted by the virus and to decipher the host immune response because of the functional diversity and highly complex anatomical and cellular composition of the CNS [20,21]. Thus, it is important to classify the cell types present in the fish brain in order to understand the molecular mechanisms involved in viral pathogens of the CNS.

Single-cell RNA sequencing (scRNA-seq) have been applied to investigate the immune system under physiological and pathological conditions [22–25]. The method provides a detailed view of the complicated immune system at single-cell resolution [26–28]. The scRNA-seq is a particularly powerful tool for identifying viral target cells, as it allows effective analysis of viral mRNAs and host signature genes in a single cell [29–32]. In addition, it can provide an unbiased characterization of virus-host interactions in individual cells, which are masked at the population level [29,30,33–38].

In this study, we profiled the deep transcriptomes of tens of thousands of individual midbrain cells of orange-spotted grouper (*Epinephelus coioides*) harvested from control and red spotted grouper NNV (RGNNV) infected fish. We determined the transcriptional profiles of immune cells to identify those enriched in infected cells and their potential functions. Our data also indicated which cell types were targeted by RGNNV infection and the activity of specific host cell genes and pathways. The extensive datasets generated during this study will be a useful resource for detailed examination of the grouper midbrain and can serve as the basis for further research on how viruses infect the teleost CNS.

## Results

### Main infection and replication sites of RGNNV in grouper brain tissue

**Fig 1:**
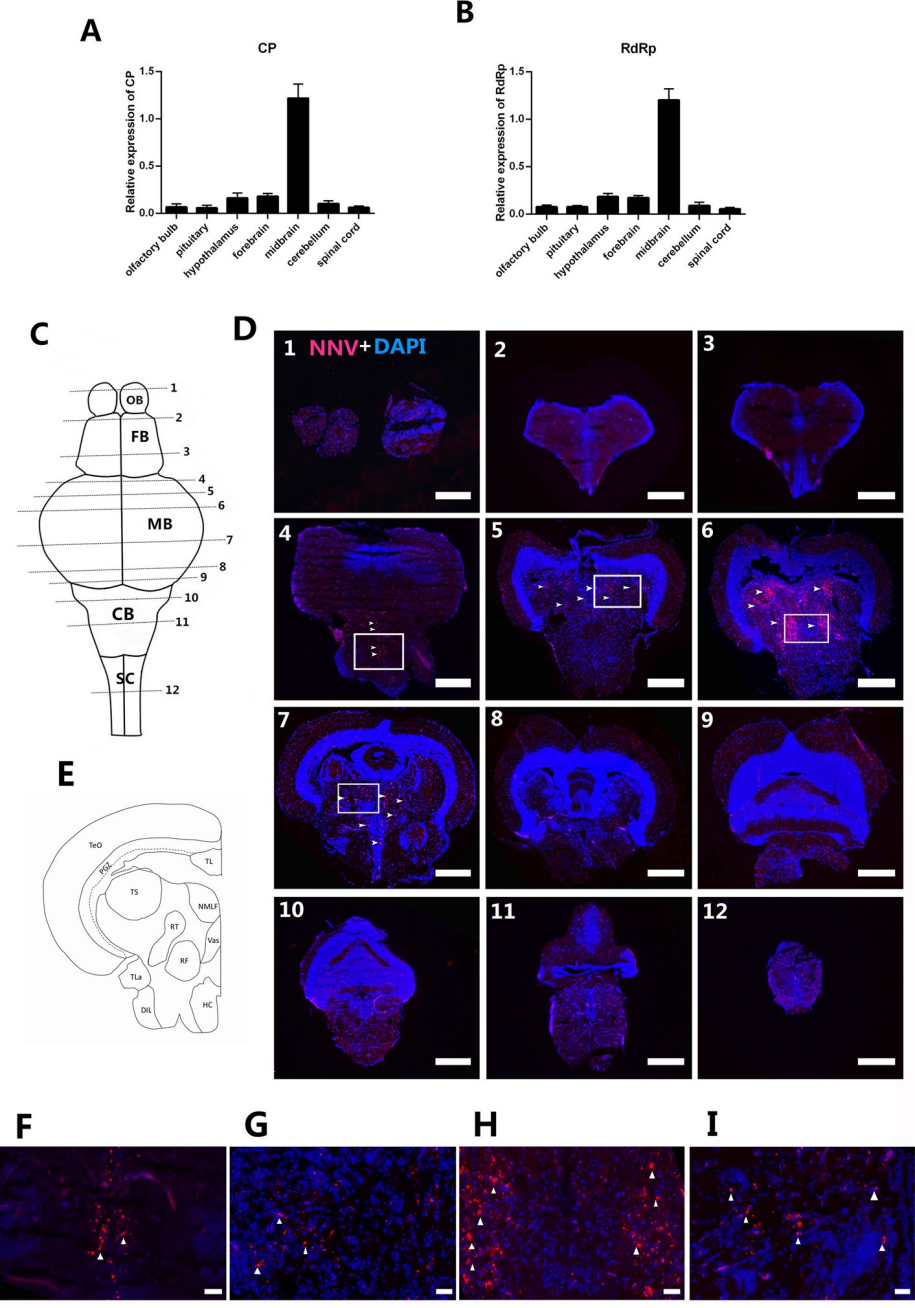
The main attack site of RGNNV in the grouper brain tissue. After infection with RGNNV, the relative quantities (compared to β-actin) of viral genomic RNA of CP (A) and RdRp (B) in different brain regions were examined using RT-qPCR (n = 5). (C) Dorsal view of the grouper brain; the horizontal line represents the slice position. (D) Localization of RGNNV as indicated by in situ hybridization. The NNV-positive signals were mainly found in midbrain (MB) regions, and the mainly replicated intercept of the MB was the position of slices 4, 5, 6, and 7. The most replicated areas were the torus semicircularis (TS), rostral tegmental nucleus (RT), reticular formation (RF), nucleus of the medial longitudinal fascicle (NMLF), and vascular lacuna of area postrema (Vas). The white arrow indicates positive staining for a probe targeting the CP region of the RGGNV genome. (E) Schematic MB drawing of the grouper. (F–I) high magnification of the boxed areas in D 4–7. OB: olfactory bulb, FB: forebrain, CB: cerebellum, SC: spinal cord, TeO: tectum opticum, PGZ: periventricular gray zone of optic tectum, TL: torus longitudinalis, TLa: torus lateralis, DIL:diffuse nucleus of the inferior lobe, HC: caudal zone of periventricular hypothalamus. Scale bars of D, 400 μm. Scale bars of F-I, 50 μm.

To identify areas of the brain that are damaged by RGNNV, we performed real-time quantitative polymerase chain reaction (RT-qPCR) and fluorescent in situ hybridization (FISH) to measure RGNNV content in different brain regions. The grouper (*E*. *coioides*) brain was divided into 5 parts of olfactory bulb (OB), pituitary, hypothalamus, forebrain (FB), midbrain (MB), cerebellum (CB), and spinal cord (SC). The expression levels of capsid protein (CP) and RNA-dependent RNA polymerase (RdRp) were significantly higher in the MB than in other brain regions (Fig 1A and 1B). We generated tissue slices of the entire grouper brain and detected the replication of RGNNV in each brain region using FISH. CP-positive signals were mainly found in the MB (Fig 1C and 1D), which indicated that this part of the brain was the main point of RGNVV infection (Fig 1D5–D7). The most infected areas were the torus semicircularis (TS), rostral tegmental nucleus (RT), reticular formation (RF), nucleus of the medial longitudinal fascicle (NMLF), and vascular lacuna of the area postrema (Vas) (Fig 1F–1I).

### Overview of the cell types in the midbrain identified by scRNA-seq

**Fig 2:**
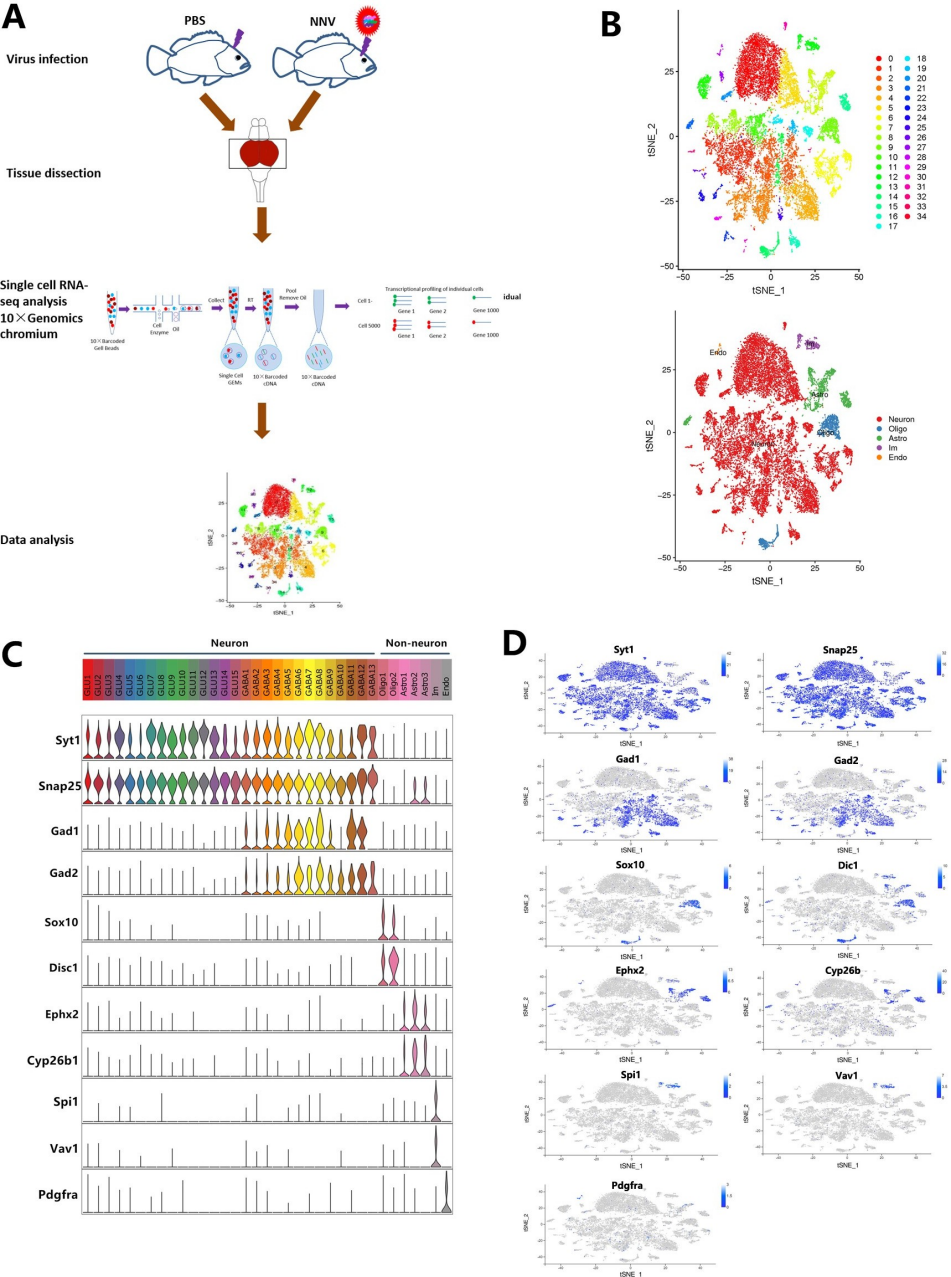
Cell sorting and categorization of cell types in the grouper midbrain. (A) Overall strategy for cell sorting and single-cell data analyses. (B) The tSNE plot clustering of cells (top): different cell clusters are color coded; in the tSNE plot showing expression of pan marker genes in distinct cell clusters (bottom), the gene expression level is color coded. (C) Violin plot showing the expression of pan marker genes across the 35 cell clusters. Each cluster is color coded. Snap25 and Syt1, pan-neuronal markers; Gad1 and Gad2, GABA neuronal markers; Sox10 and Disc1, oligodendrocyte (Olige) markers; Ephx2 and Cyp26b1, astrocyte (Astro) markers; Spi1 and Vav1, immunology (Im) cell markers; Pdgfra, endothelial (Endo) cell marker. (D) tSNE plots showing expression of pan marker genes in distinct cell clusters. The gene expression level is color coded.

Because we observed that RGNNV mainly infected grouper midbrain regions, we performed scRNA-seq on midbrain samples from uninfected and RGNNV-infected fish to characterize cellular heterogeneity in the midbrain (Fig 2A). Any cell with fewer than 400 genes or more than 10% mitochondrial unique molecular identifier (UMI) counts was filtered out, and only genes with at least one UMI count detected in at least one cell were used for further analysis. After removing cells with minimum and maximum thresholds for read numbers per cell (nUMI), number of genes detected per cell (nGene), and mitochondrial RNA genes, 13,533 and 12,464 midbrain cells were obtained from one control fish (C) and one RGNNV-infected fish (NNV), respectively (S1 Table). The nUMI, nGene, and mitochondrial mRNA percentages (pMito) are shown in S1A–S1D Fig and S1 Table. We classified cell types for all samples together and based on T-distributed stochastic neighbor embedding (tSNE) dimensionality reduction and unsupervised cell clustering. 35 cell clusters were identified based on the expressed unique transcriptional profiles as well as the top five expressed genes in each group (Fig 2B and S2 Table).

Based on the expression of the neuronal markers synaptotagmin-1 (Syt1) and synaptosomal-associated protein 25 (Snap25), the 35 cell clusters were divided into 28 neuronal (Snap25/Syt1-high) and 7 non-neuronal clusters (Snap25/Syt1 negative or low) (Fig 2C and 2D). The 28 neuronal clusters were further divided into 15 glutamatergic (GLU1–GLU15) and 13 GABAergic (GABA1–GABA18) subtypes based on the gamma-aminobutyric acid (GABA) neuronal markers Gad1 and Gad2 (Fig 2C and 2D). Among the non-neuronal clusters, there was a subset of oligodendrocyte cells with high expression of the oligodendrocyte marker genes SRY-related HMG-box 10 (Sox10) and disrupted-in-schizophrenia 1 (Disc1) (Fig 2C and 2D) as well as a subset of astrocyte cells including Astro1, Astro2, and Astro3 with high expression of the astrocyte marker genes epoxide hydrolase 2 (Ephx2) and cytochrome P450 family 26 subfamily B member 1 (Cyp26b1) (Fig 2C and 2D). There also was a subset of immune cells with high expression of the immune cell marker genes spleen focus forming virus proviral integration oncogene 1 (Spi1) and vav1 oncogene (Vav1) (Fig 2C and 2D) and a subset of endothelial cells with high expression of the immune cell marker gene platelet-derived growth factor receptor a (pdgfra) (Fig 2C and 2D). The gene expression heat maps for the top five marker genes were generated in the 35 clusters and the genes were identified which were significantly enriched in each of the 35 clusters (S2 Fig).

### Classification of non-neuronal cell types in the midbrain

**Fig 3:**
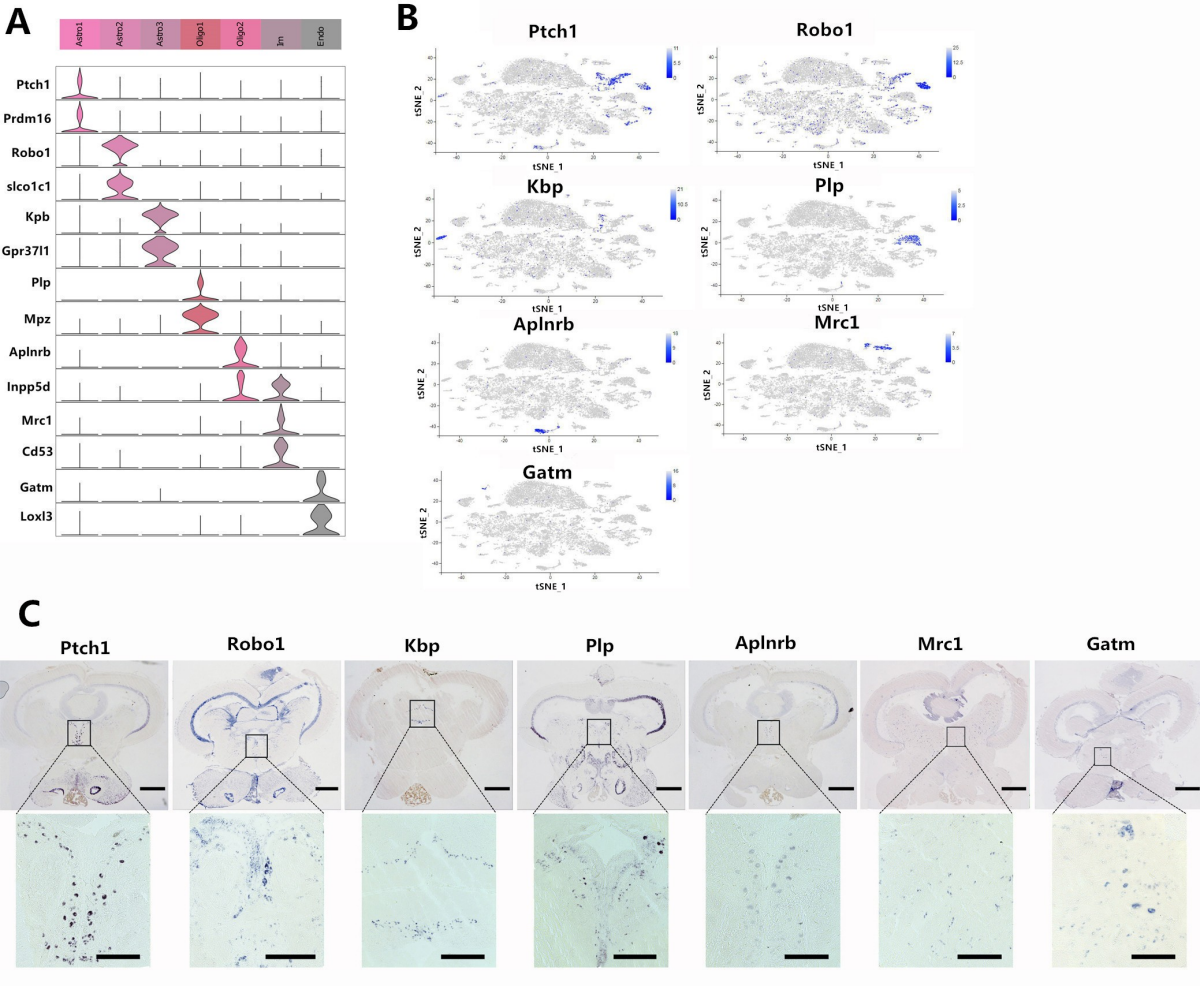
Overview of the non-neuronal cell clusters in the midbrain. (A) Violin plot showing the expression profile of representative marker genes in the seven non-neuronal cell clusters. Different clusters are color coded. Astro, astrocyte cell; Olige, oligodendrocyte cell; Im, immunology cell; Endo, endothelial cell. (B) The tSNE plots showing the expression of representative marker genes are restricted to specific non-neuronal clusters among all of the cells. The expression level is color coded. See also S3 Fig. (C) ISH data showing the expression of non-neuronal subtype markers Ptch1, Robo1, Kbp, Plp, Aplnrb, Fyb1, and Gatm in the midbrain. Above: the coronal sections of the entire midbrain region, Scale bar, 500 μm. Below: enlarged images of the regions in black squares. Scale bar, 200 μm.

The marker genes were identified for each of the seven non-neuronal clusters. Endothelial cells were marked by glycine amidinotransferase (Gatm) and lysyl oxidase-like 3 (Loxl3) (Figs 3A and 3B and S3), and Im cells were marked by CD53 antigen (Cd53) and FYN-binding protein 1 (Fyb1) (Figs 3A and 3B and S3). The two Sox10^+^ cell clusters indicative of oligodendrocytes could be distinguished from one another by subtype markers myelin protein zero (Mpz) and pyridoxal phosphate binding protein (Plp) (Oligo1) and inositol polyphosphate-5-phosphatase D (Inpp5d) and apelin receptor b (Aplnrb) (Oligo2) (Figs 3A and 3B and S3). The three Cyp26b1^+^ astrocyte clusters could be distinguished from each other by the expression of protein patched homolog 1 (Pthc1) and PR domain zinc finger protein 16 (Prdm16) (Astro1), roundabout guidance receptor 1 (Robo1) and solute carrier organic anion transporter family member 1C1 (Slco1c1) (Astro2), and kinase binding protein (Kpb) and prosaposin receptor GPR37L1 (Gpr37l1) (Astro3) (Figs 3A and 3B and S3). The tSNE plots showed that Astro1 and Astro2 were grouped together, suggesting much more subtle differences in gene expression between these subtypes (Fig 3B). Astro3 formed distinct clusters, suggesting distinct molecular fingerprints (Fig 3B).

The expression of the non-neuronal subtype markers in grouper midbrain was confirmed by in situ hybridization (ISH) (Fig 3C). Even though the tSNE plot showed that Astro3 (kbp) was far removed from Astro1 (pthc1) and Astro2 (Robo1), the ISH results showed that the kbp marker gene of Astro3 was expressed in the same region as Astro1 and Astro2, indicating that all three kind of cells may be the same type of cell. The Oligo 1 Plp^+^ was mainly localized in the periventricular gray zone of the optic tectum (PDZ), RF, and NMLF areas (Fig 3C), and the Oligo2 Aplnrb^+^ was mainly localized in the PDZ and NMLF regions (Fig 3C). This indicated that Oligo1 and Oligo2 may be the same type of cell but that there were differences between them, and our marker genes distinguished between them.

### Subpopulations of astrocytes and oligodendrocytes

**Fig 4:**
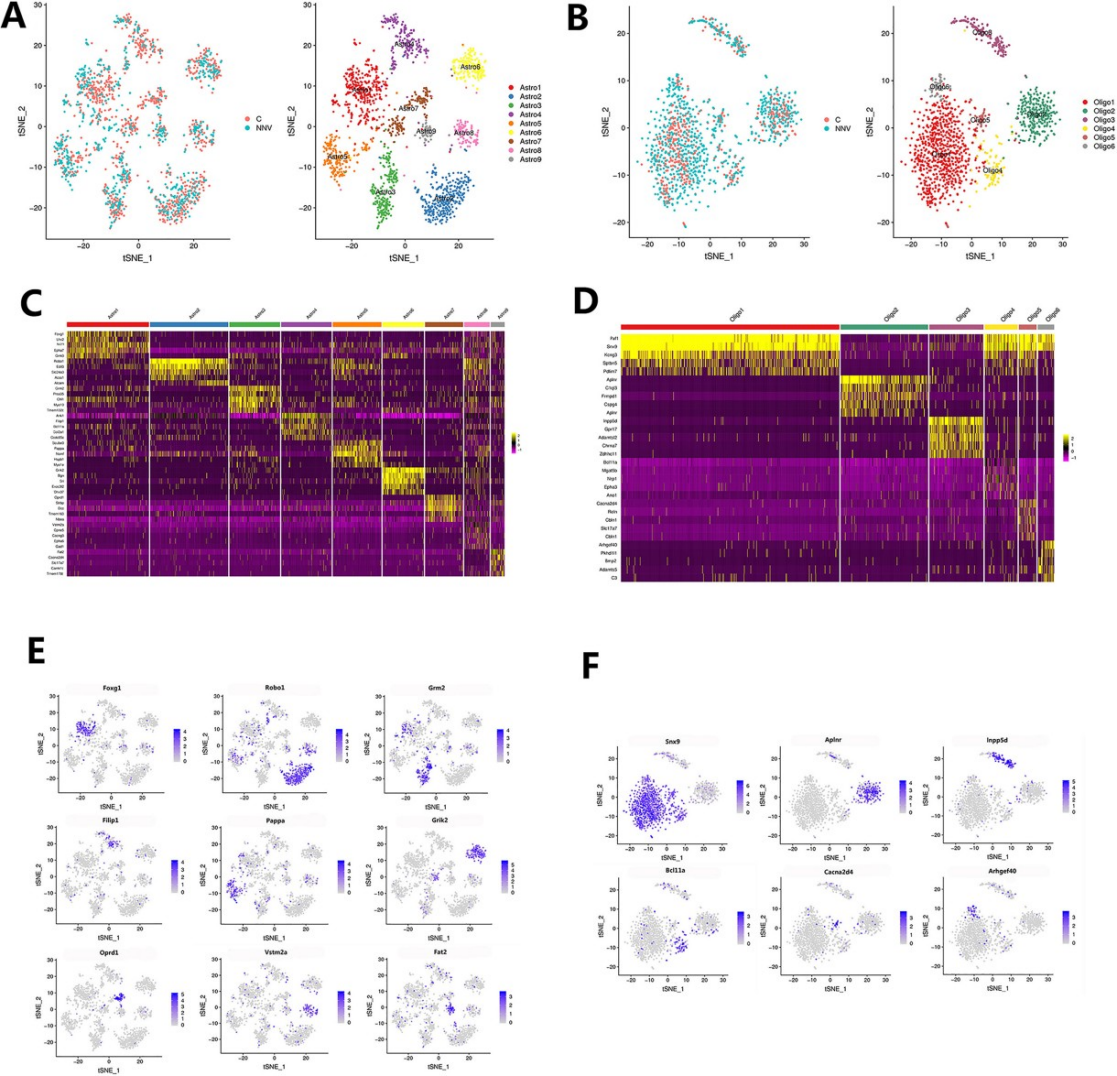
Astrocyte and oligodendrocyte subtypes in the midbrain. (A) The tSNE plot showing the nine astrocyte subtypes identified in the midbrain. Different astrocyte subtypes are color coded. (B) The tSNE plot showing the six oligodendrocyte subtypes in the midbrain. Different oligodendrocyte subtypes are color coded. (C) Heat map of the top five markers for the nine astrocyte subtype clusters. (D) Heat map of the top five markers for the six oligodendrocytes subtype clusters. (E) The tSNE plots showing the expression of representative marker genes are restricted to specific astrocyte clusters among all of the astrocyte subtype cells. The expression level is color coded. See also S4 Fig. (F) The tSNE plots showing the expression of representative marker genes are restricted to specific oligodendrocyte clusters among all of the oligodendrocyte subtype cells. The expression level is color coded. See also S5 Fig.

Nine subpopulations of astrocytes were identified, and tSNE plots showed distinct clusters, which suggested distinct molecular fingerprints (Fig 4A). The gene expression heat maps were generated for the top five marker genes in nine clusters and identified the genes which were significantly enriched in each of the nine clusters (Fig 4C and S3 Table). Additionally, tSNE plots showed the expression of selected marker genes enriched in subsets of astrocytes (Fig 4E). Six subpopulations of oligodendrocytes also were identified (Fig 4B). The gene expression heat maps were generated for the top five marker genes in 6 clusters and identified the genes which were significantly enriched in each of the six clusters (Fig 4D and S4 Table). The tSNE plots showed the expression of selected marker genes which were enriched in subsets of oligodendrocytes (Fig 4F).

### Subpopulations of immune cells, compositions and functions of macrophage cell subtypes during RGNNV infection

**Fig 5:**
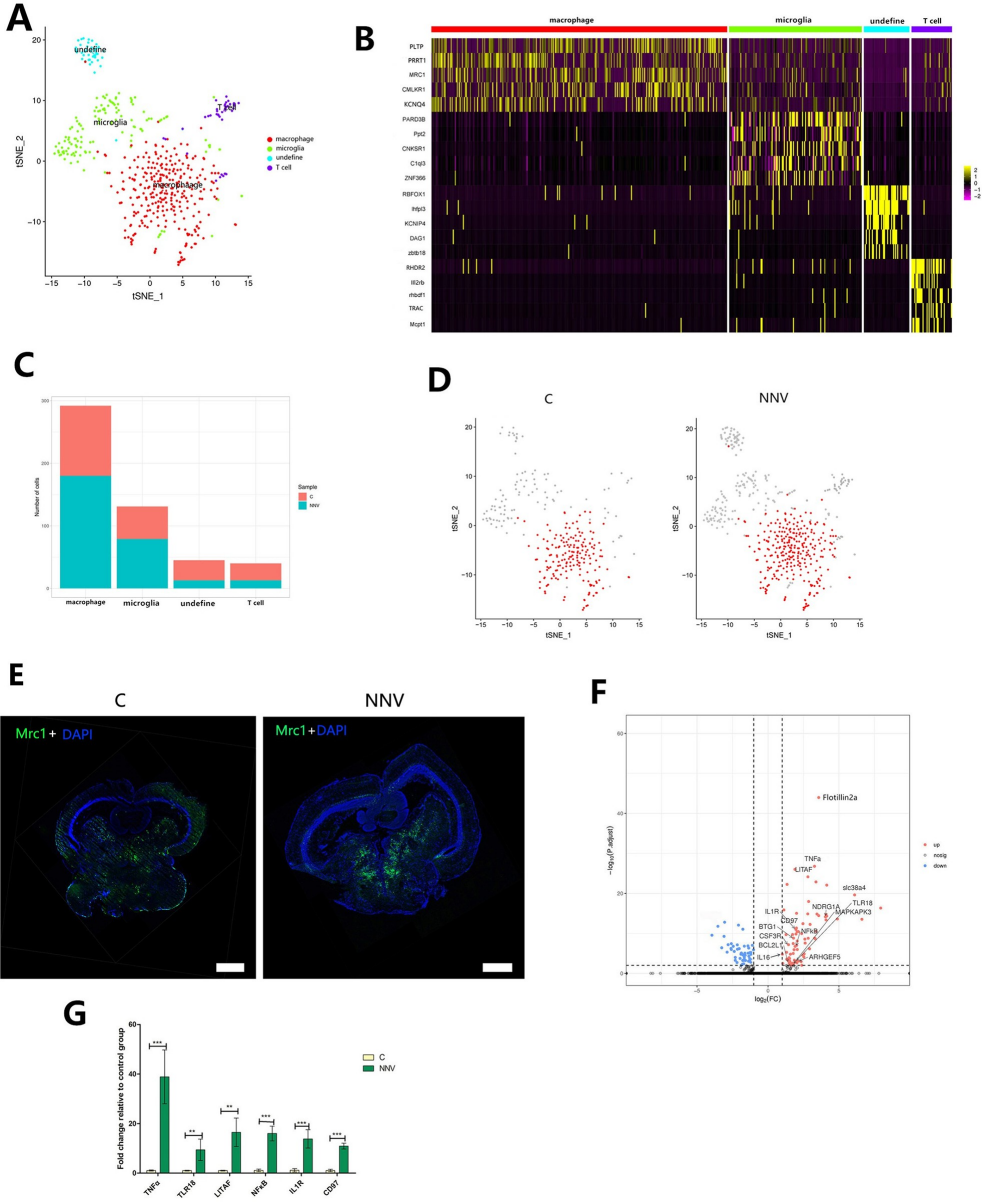
Immunological features of immune cell subsets. (A) The tSNE plot showing the four immune cell subtypes identified in the midbrain. Different immune cell subtypes are color coded. (B) Heat map showing the top five DEGs by various immune cell clusters. The gene names are listed to the left, and their corresponding cell types are at the top of the panel. (C) Number of cells in the four subpopulations of immune cells in the control and RGNNV-infected grouper. (D) The tSNE plots align the macrophage clusters between the control and RGNNV-infected grouper. (E) FISH results showing that macrophages in the midbrain were upregulated by RGNNV infection. The green stain shows the Mrc1-positive signals (macrophage marker gene), and the positive signal in the RGNNV infection group was higher than that in the control group. Scale bars, 500 μm. (F) The volcano plot shows the selected DEGs from macrophages cells in the comparison of the control and RGNNV-infected fish. DEGs of control comparison with RGNNV infection group in macrophages. Each red dot denotes an individual gene with adjust P < 0.05 (P-value adjusted by false discovery rate in MAST), other indicates non-significant genes. Example genes are labeled with the gene name. (G) The gene expression levels of IFN and inflammation-related genes increased in macrophages after RGNNV infection. Data are expressed as means ± SD. (**p < 0.01, or ***p < 0.001).

The gene expression heat maps were generated and identified marker genes for each of the subpopulations of immune cells. The four subpopulations of immune cells (Figs 5A and 5B and S6 and S5 Table) could be distinguished from each other by the expression of macrophage marker gene (Mrc1) for macrophages, Pard3b for microglia, Rbfox1 for undefined, and Rhdr2 for T cells (Figs 5B and S6). Among these cell types, macrophages were the major type of immune cell. The number of macrophages increased with RGNNV infection (Fig 5C and 5D and S6 Table), which confirmed the findings revealed by scRNA-seq. In the FISH probe test of the macrophages in the midbrain of control and RGNNV-injected fish, the Mrc1-positive signal was significantly increased after RGNNV infection (Fig 5E), which also was consistent with our scRNA-seq result. These findings suggested that macrophages played an important role during RGNNV infection.

To further investigate the changes in gene transcription levels of macrophages after RGNNV infection, we compared the expression patterns of genes between the control and RGNNV-infected cells. Expression of most immune-related genes was significantly up-regulated by viral infection, and these differentially expressed genes (DEGs) were involved in myeloid leukocyte activation and related to nuclear factor (NF)-κB signaling. Analysis of gene expression levels in midbrain cells from control and RGNNV-infected fish revealed that expression of most of the pro-inflammatory cytokine genes was higher in infected fish, including tumor necrosis factor alpha (TNFα), interleukin 1 (IL1), lipopolysaccharide induced TNF factor (LITAF), Toll-like receptor 18 (TLR18), CD97, and NFκB (Fig 5E). The RT-qPCR results also showed high expression of these genes after RGNNV infection (Fig 5F). Together, these findings illustrated a consistent response by innate immune cells to RGNNV infection.

### Differentiation of macrophages during RGNNV infection

**Fig 6:**
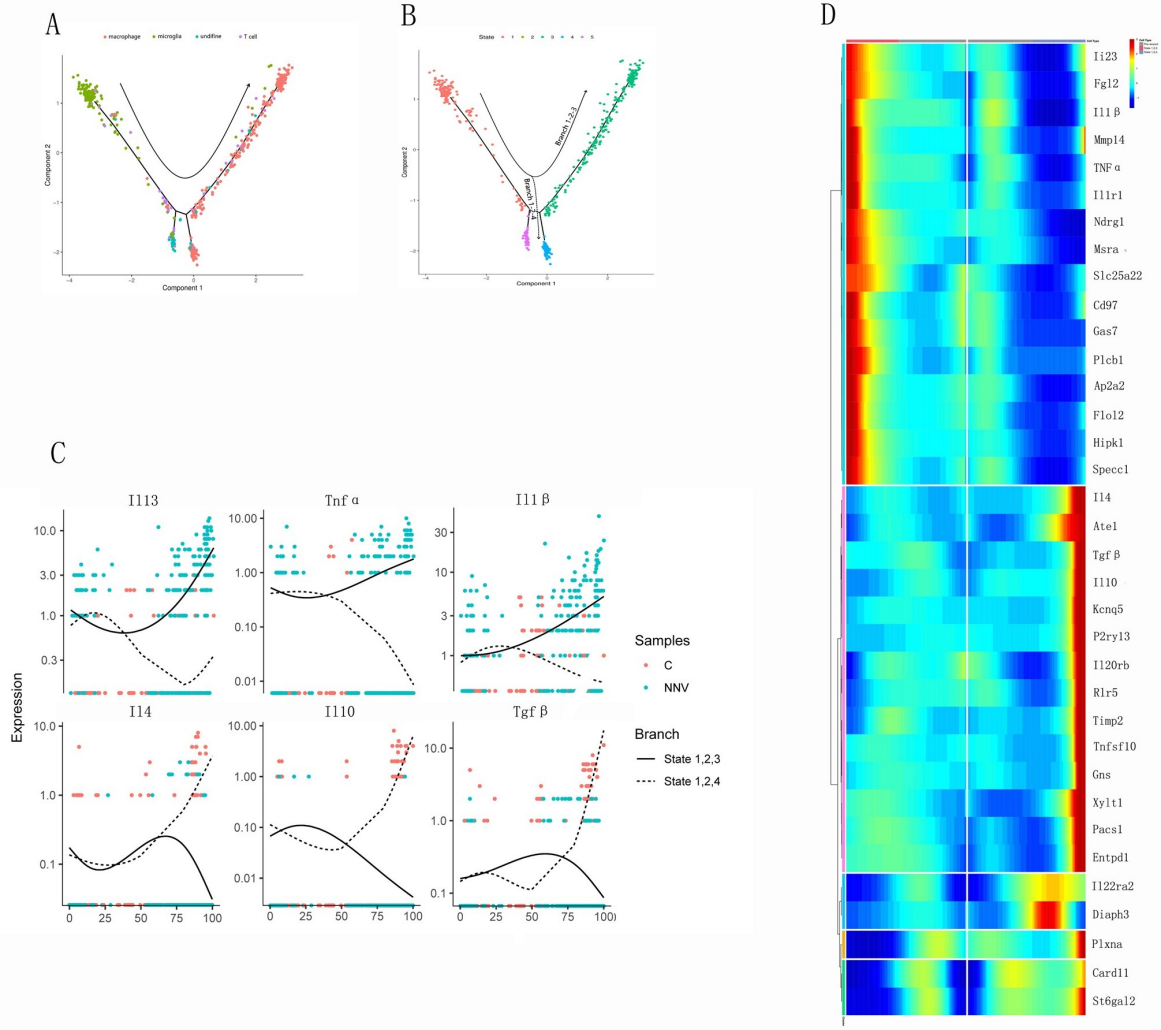
Transcriptional dynamics during development of macrophages. (A) Pseudotemporal ordering of different clusters based on their gene expression profiles. Arrows indicate the direction of differentiation. (B) Pseudotemporal ordering of differentiated states in the macrophage cells. The dashed line represents branch 1-2-3; the solid line represents branch 1-2-4. Cells are colored by state. (C) Expression of M1-type macrophage and M2-type macrophage marker genes along the two primary branches ordered in pseudotime. Solid lines represent expression in branch 1-2-3 (M1-type macrophages), and dashed lines represent expression in branch 1-2-4 (M2-type macrophages). (D) Branched heat map showing macrophages with highly significant branch-specific expression patterns in pseudotime. The root of the tree is in the middle of the plot, and expression from the earliest to the M1-type macrophages progresses to the left, whereas the progression of M2-type macrophages progresses to the right of the root.

Immune cells differentiate to resist virus invasion. For example, macrophages differentiate into M1 or M2 types to exert their immune function. To evaluate this process in macrophages during RGNNV infection, we performed an unsupervised pseudotime analysis. The main cell differentiation observed was from microglia to macrophages (Fig 6A). Furthermore, the cell trajectory branching analysis showed that Il-23, Tnf-α, and Il-1β (genes highly expressed in M1-type macrophages) were highly expressed in branch 1-2-3, but expression was significantly decreased in branch 1-2-4. Expression of Il-4, Il-10, and Tgf-β (genes highly expressed in M2 type macrophages) was significantly increased in branch 1-2-4 but significantly decreased in branch 1-2-3 (Fig 6B and 6C).

To further characterize the transcriptional program underlying macrophage differentiation, we then identified five groups of genes with distinct expression patterns along the differentiation process (Fig 6C). These results suggested that RGNNV infection may lead to the transformation of microglia into macrophages, which then mainly transformed into M1-type activated macrophages.

### RGNNV mainly attacked nerve cells during infection

**Fig 7:**
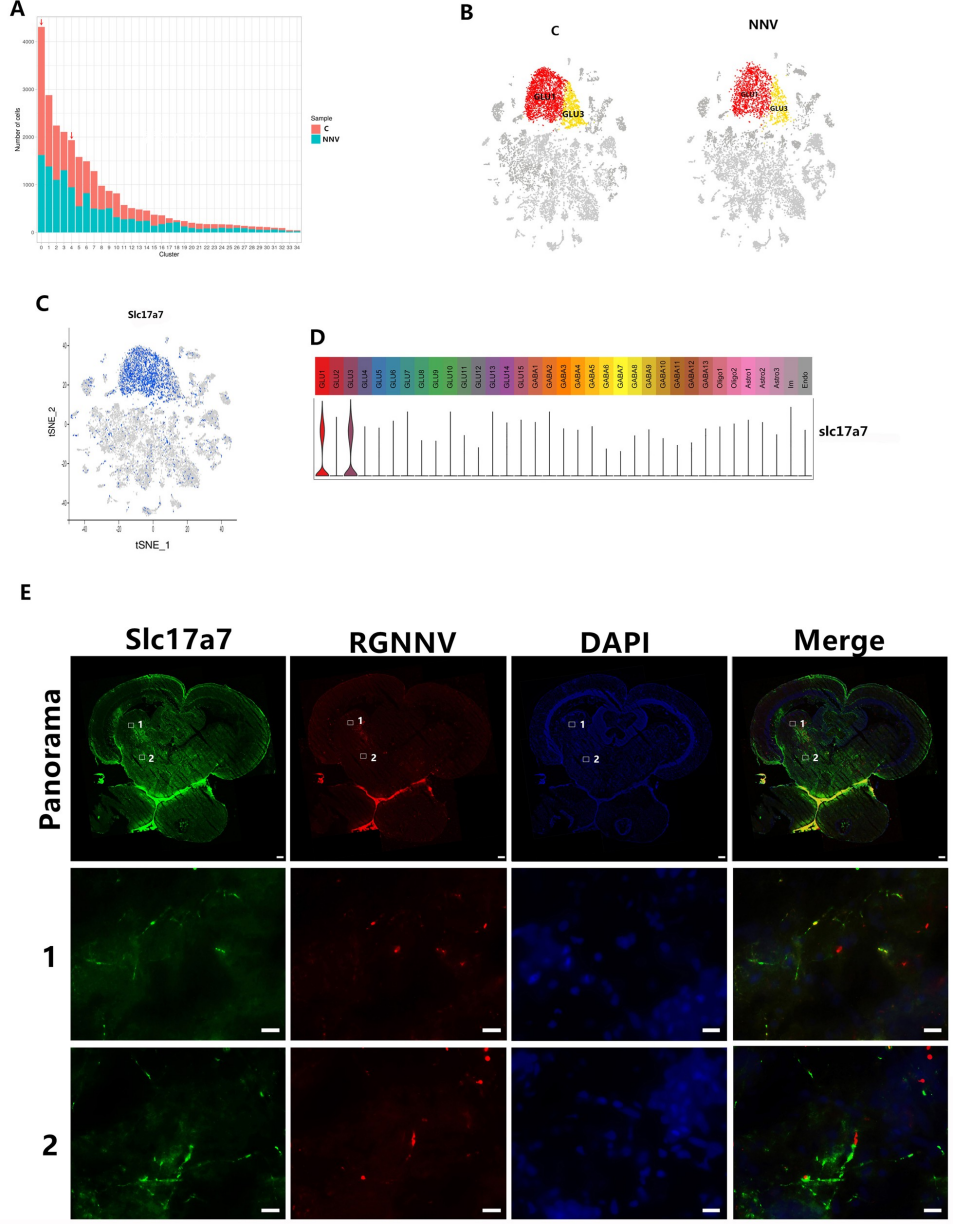
Screening of cell types attacked by RGNNV. (A) The number of cells in each cell cluster in control and RGNNV-infected grouper. (B) The tSNE plots showing the GLU1 and GLU3 clusters for control and RGNNV-infected grouper. (C) The tSNE plot showing that expression of the marker gene slc17a7 was restricted to specific GLU1 and GLU3 clusters among all of the cell clusters. (D) Violin plot showing the expression of pan marker genes across the 35 cell clusters. (E) Multicolor FISH staining to validate the Slc17a7 and RGNNV states, GLU1 and GLU3 cells were marked with Slc17a7. The middle row (1) shows high magnification of the boxed areas 1 in the top row (panorama). FISH staining shows that Slc17a7 and RGNNV were co-located in the same neuron (scale bars 10 μm). The bottom row (2) shows high magnification of the boxed areas 2 in the top row (panorama). RGNNV and Slc17A7 did not co-locate, but they localized in different areas of the same neuron (scale bars 10 μm). Scale bars in the panorama view are 200 μm.

Of the nerve cell subgroups, cell numbers in the two groups GLU1 and GLU3 were reduced the most after RGNNV infection (reduced by 40% and 47%, respectively) (Fig 7A and 7B and S7 Table). To confirm that RGNNV attacked GLU1 and GLU3 subgroup cells, FISH was used to test the co-localization of GLU1 and GLU3 nerve cells and RGNNV. Slc17a7, which was specifically highly expressed in both GLU1 and GLU3 subgroup cells, was used as the marker gene to mark GLU1 and GLU3 cells (Fig 7C and 7D). The FISH results showed that Slc17a7-positive and CP-positive signals were co-located in all five RGNNV-infected fish (Fig 7E), which indicated that RGNNV co-localized with GLU1 and GLU3 nerve cells. These results supported the premise that RGNNV mainly attacked GLU1 and GLU3 nerve cells.

**Fig 8:**
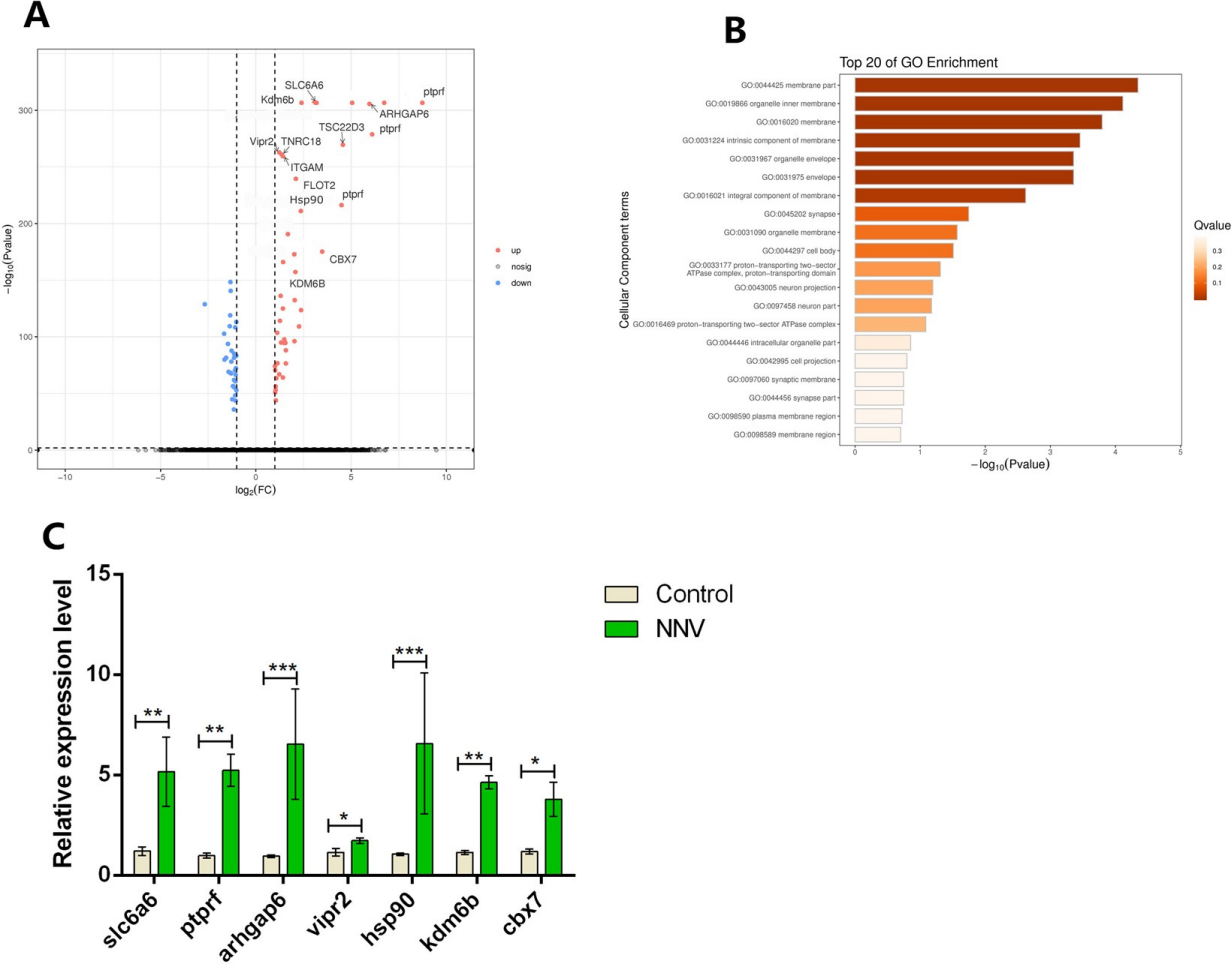
Comparison of gene expression of GLU1 and GLU3 cells between control and RGNNV-infected fish. (A) The volcano plot shows the selected DEGs of immune cells from control vs. RGNNV-infected fish. Only genes specifically upregulated in GLU1 and GLU3 cells were evaluated. Each red dot denotes an individual gene with adjusted P < 0.05 (P-value adjusted by false discovery rate in MAST), others are non-significant genes. (B) GO terms are labeled with name and ID and sorted by −log10 (P) value. The top 20 enriched GO terms are shown. (C) The genes for which expression levels increased after RGNNV infection in GLU1 and GLU3 cells. Data are expressed as means ± SD. *p < 0.05, ** < 0.01, or *** < 0.001.

To further investigate the transcriptomic changes of GLU1 and GLU3 after RGNNV infection, we compared the expression patterns of control and RGNNV-infected fish. We found that DEGs associated with membrane formation, transfer, and autophagy related pathway were significantly enriched in the RGNNV infection group, and high levels of protein tyrosine phosphatase receptor type F (ptprf), vasoactive intestinal peptide receptor 2 (vipr2), heat shock protein 90 (hsp90), lysine (K)-specific demethylase 6B (kdm6b), rho-GTPase activating protein 6 (arhgap6), and solute carrier family 6 member 6 (slc6a6) were detected (Fig 8A and 8B). The RT-qRCR results also showed that membrane receptor, membrane lysis, transmission, and autophagy related genes were highly expressed in the RGNNV infection group (Fig 8C).

## Discussion

Red spotted grouper nervous necrosis virus is a common fish pathogen that destroys the nervous system, and vacuolization of the brain is the main cause of death caused by the virus [15,16]. Identifying the specific areas and cell types in the CNS targeted by the virus is crucial for understanding the pathogenesis of RGNNV infection. However, it is difficult to obtain an integrated scenario of the cellular and molecular immune responses that occur upon RGNNV infection. To address this issue, we performed scRNA-seq and identified 35 transcriptionally distinct cell subtypes in the grouper midbrain, including 28 neuronal and seven non-neuronal cell types. Analysis of the transcriptional profiles of immune cells revealed macrophage enrichment in RGNNV-infected fish, and the macrophages were mainly polarized into M1-type macrophages and showed a strong inflammatory response. We also found that RGNNV mainly targeted GLU1 and GLU3 nerve cells and that RGNNV infection induced significant enrichment of membrane formation, lysis, and vesicular transport related pathways. These results provided a comprehensive overview of midbrain cell types based on their transcriptional features, identify cell types involved in the antiviral response, and revealed which cell types are targeted by RGNNV.

Researchers previously conducted a detailed census of cell types in the nervous system of mice [39,40], and cell types in part of the brain of reptiles also have been reported [41]. However, the types of cells in the brain of fish are poorly known, even though fish play an important role in the evolution of vertebrates. In this study, we using RGNNV infected young orange-spotted grouper (*E*. *coioides*) (mean weight 3.3 ± 0.4 g, mean body length 3.7 ± 0.3 cm) and obtained 13,533 and 12,464 cells from one control fish (C) and one RGNNV-infected (NNV) fish midbrain after single cell collection. Also, we identified cell type-specific markers to allow for unambiguous cell type definition, which will enable the development of genetic or viral tools to achieve cell type-specific labeling and manipulation. This ability is essential for analysis of cell type-specific functions in the complex midbrain region. Major classes of cells, such as neurons, astrocytes, endothelial cells, oligodendrocytes, and immune cells, can be distinguished by large sets of class-specific genes that are related to the specific function of each class of cells. In mammals and reptiles, cells in different brain regions can be divided into neuronal and non-neuronal clusters based on the expression of the pan neuronal markers Snap25 and Syt1, and neuronal clusters can be further divided into glutamatergic (GLU) and GABAergic (GABA) subtypes based on the GABA expression of Gad1 and Gad2 [41,42]. We found the neuronal markers Snap25 and Syt1 in 28 clusters, which in turn could be divided into 15 GLU (GLU1–GLU15) and 13 GABA (GABA1–GABA13) subtypes based on their differential expression of Gad1 and Gad2. This indicated that the mammalian nervous system divisions also applied to fish [43]. We also identified potential subtype-specific marker genes for each of the 28 neuronal clusters (S2 Table). The majority of them contained subtype-specific genes unique to that cluster, but in some cases a neuron cluster could be defined by the combinatorial expression of marker genes (Fig 2C and S2 Table). We distinguished a number of neuron subtypes by the expression of specific genes. For example, Cbln1 and TBR1 were only found in Glu1 and Glu4 cell clusters, respectively (S2 Table). Some genes were expressed only in certain types of nerve cells. Lhx9 was only expressed in Glu nerve cells, such as Glu6, Glu2, Glu5, and Glu13 (S2 Table). These results demonstrated that our unbiased scRNA-seq analyses were able to identify cell types as well as cell type-specific transcriptional features in the midbrain.

In mammals, the nervous system tends to consist of both nerve cells and non-nerve cells, and non-nerve cells mainly fall into four categories according to special marker genes: astrocytes, oligodendrocytes, immune cells, and endothelial cells [40]. The types of non-nerve cells in the fish nervous system were previously unknown. Based on non-neuronal clusters marker genes in mammals and amphibians, we identified four non-nerve cell types in grouper: astrocytes (Ephx2^+^ and Cyp26b1^+^), oligodendrocytes (Sox10^+^ and Disc1^+^), immune cells (Spi1^+^ and Vav1^+^), and endothelial cells (Pdgfra^+^) (Fig 2) [39–42]. However, we also identified unique non-nerve cell marker genes in the grouper midbrain. For example, based on grouper-specific gene expression, astrocytes were divided into three clusters [Astro1 (ptch1^+^ and prdm16^+^), Astro2 (robo1^+^ and slco1c1^+^), and Astro3 (kpb^+^ and gpr37l1^+^)] and oligodendrocytes were divided into Oligo1 (plp^+^ and mpz^+^) and Oligo2 (aplnrb^+^ and inpp5d^+^). The expression of some non-neuronal subtype markers in the grouper midbrain were confirmed by ISH with riboprobes that were synthesized according the marker gene sequence (S1 Supplementary Sequence) of each non-neuronal subtype. These results suggested that the classification of CNS cells in the fish midbrain was comparable to that of other vertebrates, but the orange-spotted grouper also had its own unique marker genes.

The host immune response against acute RGNNV infection plays an antiviral role but also leads to simultaneous pathogenic injury to organs and tissues, especially in the brain of grouper. Several researchers have reported the characteristics of innate and adaptive immune responses after virus infection [43–48] which has allowed us to speculate about the potential pathogenesis of RGNNV infection. However, it is difficult to obtain an integrated scenario of the cellular and molecular immune responses in fish infected by RGNNV. To address this issue, we profiled the immunological response landscape in RGNNV-infected grouper at single-cell resolution to identify the critical factors responsible for antiviral immunity and pathogenesis in infected fish. We identified expanded effector macrophage clusters, which may provide a possible immune response in RGNNV-infected fish. We also found that RGNNV-infected grouper showed a concerted and strong inflammatory response. Additionally, compared to control fish, viral infection led to changes in host cell transcription of several inflammatory cytokines, including TNFα, IL1, LITAF, TLR18, and NFκB. Previous studies of RGNNV showed that the virus could harness the inflammatory response to antagonize innate immunity in *in vitro* cell infection experiments [49–51]. In fish, interferon (IFN) is an important barrier against viral invasion [52–54]. However, none of the IFN-related genes was significantly overexpressed after RGNNV infection. Studies have shown that many RNA viruses could evade the host’s immune response. For example, the rabies virus can prevent the acidification of IRF3 by TBK1 [55], Ebola virus can inhibit the activation of IRF3 [56], and porcine reproductive and respiratory syndrome virus suppresses type I IFN production and signaling, manipulates the cytokine responses, and modulates apoptosis to establish persistent infection in pigs [57]. A recent study in grouper spleen cells (GS) demonstrated that the B2 protein of RGNNV could inhibit the host IFN response by suppressing host transcription directed by RNA polymerase II [58]. This suggests that RGNNV may have a potential mechanism to evade the host immune response. Based on our data, we confirmed that RGNNV infection caused a concerted and strong inflammatory response *in vivo*, and we identified the inflammatory cytokines with high expression during RGNNV infection. However, the state of the immune system was not fully restored, as exemplified by the IFN-related subset. A long-term follow-up study is needed to investigate how fish achieve full immunity to persistent RGNNV.

In fish, macrophages are a key component of the innate immune system. Activated macrophages can produce active molecules to guide the occurrence and regression of inflammation, so they are crucial for antivirus activity and survival of organisms [59]. Microglia cells are widely considered to be immune effectors in the CNS and to play an important role in the treatment of the injured CNS. However, it is not clear whether they have any effect on the nervous system damage caused by the virus’s attack [60]. Our results showed that microglia cells could be transformed into M1-type activated macrophages after viral invasion of grouper brain tissue. Studies have shown that microglia can activate macrophages to express cytokines such as IL-1 to reduce neuron damage [61,62]. We also found that IL-1 levels were significantly increased in brain macrophages of RGNNV-infected grouper. These results suggested that viral attack of nerve cells might stimulate microglia cells to transform into M1-type activated macrophages to produce cytokines to reduce the damage to nerve tissue caused by the virus.

Previous studies reported that RGNNV mainly attacked grouper nerve cells, leading to fish death, but it was not clear which types of nerve cells are targeted by RGNNV. Previous studies also showed that the viral infection did not lead to cells being differentially excluded from the scRN-seq dataset [63–65]. Thus, in the present study we used scRNA-seq to divide nerve cells into 28 classes, and we compared control and RGNNV-infected fish to determine which cell types were lost due to virus infection. Our results showed that the number of GLU1 and GLU3 nerve cells was reduced seriously (decreases of 39.58% and 46.80%, respectively) in infected fish. Furthermore, the marker genes of GLU1/GLU3 nerve cells and virus CP genes were co-localized, which illustrated that they were the main nerve cells attacked by RGNNV. Further analysis of the genes in the GLU1/GLU3 cells that were differentially expressed between the control and RGNNV-infected fish indicated that many receptor genes were highly expressed, and these receptors may be receptors for RGNNV. Like the significantly overexpressed gene HSP90, which is a receptor for Dengue virus and Japanese encephalitis virus [66,67], many other receptor genes, such as Ptprf and Vipr2, were significantly highly expressed in grouper after RGNNV infection. However, identifying which receptor is the RGNNV receptor requires further study.

Previous study reported that RGNNV infection grouper spleen cells (GS) cell resulted in the fusion and enlargement of numerous cytoplasmic vacuoles [68]. However, the origin and mechanism of cytoplasmic vacuolization remain unknown. The vacuolization effects caused by viral infection have been investigated in members of 15 viral families, including hepatitis A virus, hepatitis C virus, bovine virus diarrhea virus, murine leukemia virus, Zika virus, hepatitis B virus, and polyomaviruses [69,70]. Although the origin of virus-induced vacuoles has not been fully characterized, several reports demonstrated that the mechanisms underlying the virus-induced vacuolization effects differed among viruses. For example, 3C protease of hepatitis A virus induced numerous non-acidic cytoplasmic vacuoles, which originated from the endosome and lysosome compartments [71]. Simian virus 40 was found to induce substantial cytoplasmic vacuoles at the late productive infection stage, and binding of the viral major capsid protein VP1 to the cell surface ganglioside, GM1, triggered the formation of cytoplasmic vacuoles [72,73]. All of these studies indicated that virus-induced vacuoles derived from different membrane organelles, including mitochondria, endoplasmic reticulum, lysosomes, Golgi apparatus, and autolysosomes. In this study, we showed that RGNNV targeted GLU1/GLU3 nerve cells, and we found that pathways related to membrane formation, lysis, and transmission were significantly enriched in these two cell types. Additionally, cytoplasmic vacuole formation and apoptosis-related genes were significantly highly expressed in these cell types after RGNNV infection. For example, lysine (K)-specific demethylase 6B (Kdm6b) was the most important gene involved in the induction of neuro-dysmorphia and it promotes cell apoptosis [74,75]. Furthermore, rho-GTPase activating protein 6 (Arhgap6) is an important gene for cytoskeleton rearrangements and may play an important role in cytoplasmic vacuole formation [76]. Both genes were significantly highly expressed after RGNNV infection, which indicated that they may had important functions in the death of GLU1/GLU3 nerve cells. Additionally, Kaul and Lipton showed that HIV could induce injury and apoptosis in rodent and human neurons *in vitro* and *in vivo* via the release of macrophage toxic factors [77]. We found that the number of macrophages increased significantly in grouper after RGNNV infection, thus we speculated that nerve cell death may also be induced by toxic factors released by macrophages, but further research is needed.

In summary, this is the first report of the cell types present in the grouper midbrain. It provided a comprehensive transcriptional perspective of the fish midbrain, which will be a valuable resource for identifying cell type-specific functions of this complex brain region. Using scRNA-seq analysis, we demonstrated many new phenomena involved in the pathogenesis of RGNNV infection in grouper. We found that macrophages may be the key cell type required for elimination of RGNNV. Furthermore, we speculated that microglia may differentiate into M1-type activated macrophages, and this may be an immune response of grouper during RGNNV infection. Based on the analysis of DEGs of macrophages cells, we also speculated that cytokines may contribute to the persistence of RGNNV. Finally, we found that RGNNV attacked GLU1 and GLU3 cells, and we screened for genes that cause GLU1 and GLU3 cell vacuole morphology changes and death. Results of this study provided greater understanding of how fish viruses attack the nervous system and how the CNS resists viruses.

## Materials and methods

### Ethics statement

All animal experiments were carried out in strict accordance with the guidelines and regulations of the Animal Research and Ethics Committees of South China Agriculture University (SYXK-2019-0136).

### Animals and RGNNV infection

Two hundred orange-spotted grouper (*E*. *coioides*) (mean weight: 3.3 ± 0.4 g, mean body length: 3.7 ± 0.3 cm) were used in this experiment. Fish were maintained at 25–30°C in fresh seawater, which was disinfected and sterilized. The fish were fed a commercial diet according to a standard feeding scheme. For the RGNNV treatment, 50 fish were placed in each of two tanks and injected intraperitoneally with 20 μL of 107 TCID50/ml RGNNV (NNV group hereafter). For the control group (C), 50 individuals were placed in each of the two tanks and injected with 20 μL of phosphate buffered saline (PBS). when more than 50% of the fish in the virus injected group showed clinical signs of disease one control and one diseased fish were sacrificed, and midbrain were collected for single cell RNA-Seq analysis. At the same time 15 control fish and 15 diseased fish were sacrificed, and each brain was dissected and fixed in 4% buffered paraformaldehyde overnight at 4°C, rinsed twice with cold PBS, transferred to 30% sucrose solution for 48 h, and stored at 4°C. The samples dried with sucrose were frozen using dry ice and embedded in optimal cutting temperature (OCT) compound (Sakura, USA) and used for FISH analysis. The remaining fish (eight control and 15 showing typical disease signs) were sacrificed and whole midbrain (approximately 30 mg) tissues were frozen in liquid nitrogen immediately and stored at -80°C before RNA extraction. This work received approval from the Animal Research and Ethics Committees of South China Agriculture University (SYXK-2019-0136).

### Single-cell collection and cDNA amplification

Single-cell capture was performed using a Chromium Controller instrument (10x Genomics, Pleasanton, CA, USA), which is a highly repeatable, efficient, and stable device for cell characterization and gene expression profiling of thousands to millions of cells (https://www.10xgenomics.com/solutions/single-cell/). Single cells were collected from the whole midbrain (approximately 30 mg) of one control fish and one RGNNV-infected fish showing typical disease signs of disease as follows. Midbrain tissue was transferred from the cryopreserved tube to a Dounce homogenizer, and 500 μL of precooled cracking buffer were added. The nuclei extraction was perform as described previously [78]. Briefly, after homogenizing the tissue, the homogenate was filtered through a 70 μm cell screen, and iodoxanol gradient solution was added prior to centrifugation. The obtained white nuclear layer was washed with nuclear cleaning buffer. After trypan blue staining, the nuclear suspension was assessed using a cell counting plate under a microscope, and the total amount, concentration, and nuclear ratio of cells with an intact nuclear membrane were calculated. The target concentration of the nuclear suspension was 700–1,200 nuclei/μL. Nucleus suspensions were loaded on the Chromium Controller instrument to generate single-cell gel bead-in-emulsions (GEMs) using Chromium Single Cell 3’ Reagent v3 Kits (10× Genomics) containing a pool of ~750,000 barcodes sampled to separately index the transcriptome of each cell. Thousands of individual cells were isolated into droplets together with gel beads coated with unique primers bearing 10× cell barcodes, unique molecular identifiers (UMI), and poly (dT) sequences. According to the single cell 3’ reagent kit protocol, GEM-reverse transcriptions were performed in a Veriti 96-well thermal cycler (Thermo Fisher Scientific, Waltham, MA, USA). After reverse transcription, GEMs were broken and the barcoded single-strand cDNA was cleaned up using DynaBeads MyOne Silane Beads (Thermo Fisher Scientific) and a SPRI Select Reagent Kit (Beckman Coulter, Brea, CA, USA). Global amplification of cDNA was achieved using the Veriti 96-well thermal cycler, and the amplified cDNA product was cleaned up using the SPRIselect Reagent Kit.

### Library construction and sequencing

The indexed sequencing libraries were constructed using the reagents in the Chromium Single Cell 3’ Library v3 Kit for fragmentation, end repair, and A-tailing; size selection with SPRI select beads; adaptor ligation; post ligation cleanup with SPRI select beads; and sample index PCR and final cleanup with SPRI select beads. The final single cell 3’ library contained the standard Illumina paired-end constructs which begin and end with P5 and P7 primers used in Illumina bridge amplification. The barcoded sequencing libraries were quantified using a Bioanalyzer Agilent 2100 System with a High Sensitivity DNA chip (Agilent, Santa Clara, CA, USA) and quantitative PCR using a KAPA Library Quantification Kit (KAPA Biosystems). Finally, two sequencing libraries were loaded onto a HiSeq2500 (Illumina, San Diego, CA, USA) with a custom paired-end sequencing mode (26 base pairs for read 1 and 98 base pairs for read 2).

### Initial quality control

The single-cell sequencing files (base call files) were processed using the Cell Ranger Single-Cell Software Suite (v2.0) for quality control, sample demultiplexing, barcode processing, and single-cell 3’ gene counting [79]. The raw base call files for each sample were first multiplexed into fastq data using bcl2fastq conversion software. In the raw data analysis, the fastq data were aligned to the orange-spotted grouper genome sequence (china national genebank:CNA0000026, https://db.cngb.org/search/assembly/CNA0000026/) using STAR with default parameters. Quality control of the fastq data was performed using FastQC software, and the data were aligned to the Nucleotide Sequence Database (https://www.ncbi.nlm.nih.gov/genbank/) using the basic local alignment search tool (BLAST) to avoid the data distortion caused by experimental contamination by other species, especially bacterial infection or contamination. After the initial quality control, the sequences with low quality barcodes and UMIs were removed.

For further counting of the UMI tags, the CellRanger count algorithm was used to generate single-cell gene counts for a single library. This approach can provide the most stable and accurate clustering solutions for 10x Genomics scRNA-seq data [80].

Only confidently mapped, nonPCR duplicates with valid barcodes and UMIs were used to generate the gene-barcode matrix. To quantitatively identify intracellular viral segmented mRNAs to track the cells from the midbrain of fish infected with RGNNV at single-cell resolution, the scRNA-seq data of two midbrain samples were reanalyzed using the CellRanger count algorithm based the union of the mm10 and PR8 (txid211044, NCBI) reference genomes. To compare the scRNA-seq data among different libraries, the gene-cell-barcode matrix of each sample was normalized by equalizing the read depth between libraries for further merging using the CellRanger aggregate procedure, which was confirmed using the Seurat integrated analysis method [81].

The reads from higher-depth libraries were subsampled until all libraries had an equal number of confidently mapped reads per cell. The gene-cell-barcode matrix from each of the two midbrain samples was concatenated, log-transformed, and filtered based on the number of genes detected per cell. Any cell with fewer than 400 genes or more than 10% mitochondrial UMI counts was filtered out, and only genes with at least one UMI count detected in at least one cell were used for further analysis, which was performed using CellRanger R version 2.0.0 and Seurat suite version 2.0.0.

### Clustering, differential expression, and visualization

To cluster the cells, principal component analysis (PCA) was run on the normalized filtered gene-barcode matrix to reduce the number of feature (gene) dimensions. The top five principal components were selected and passed to t-distributed Stochastic Neighbor Embedding [82] for clustering visualization in a two-dimensional space. Graph-based clustering was then run to group cells with similar expression profiles, thereby building a sparse nearest-neighbor graph without pre-specification of the number of clusters. Clusters were grouped into 35 unsupervised categories according to the differential expression profile of hallmark genes. To identify genes that were enriched in a specific cluster, the mean expression of each gene was calculated across all cells in the cluster. Each gene from the cluster then was compared to the median expression of the same gene from cells in all other clusters, and the log_2_ fold-change of differentially expressed genes (DEGs) was calculated. For hierarchical clustering, pairwise Pearson’s correlation between each cluster was calculated based on the mean expression of each gene across all cells in the cluster, and the log_2_ fold-change of DEGs was used to create a heat map to visualize the results using MEV software (http://www.tm4.org/). Specific gene expression was graphically represented using T-distributed stochastic neighbor embedding (tSNE) plots implemented using Loupe Cell Browser software and Cell Ranger R.

### Single-cell PCA analysis and gene ontology enrichment

The transcriptional profile data for immune cell and GLU1 and GLU3 were retrieved from the NIH SRA database with the accession code SRP040656 (https://www.ncbi.nlm.nih.gov/sra/). The most marker genes used for grouper cell classification can aligned with mouse marker gene well, most of them can identity over 60% (S8 Table). After z-score normalization, the transcriptional profile data for immune cell and GLU1 and GLU3 from the database together with the specific cell clusters in our study were used for pairwise Pearson’s correlation and PCA analysis implemented in R language (http://www.r-project.org) to demonstrate the phylogeny of specific clusters. Functional pathways representative of each gene signature was analyzed for enrichment in gene categories from the Gene Ontology Biological Processes (GO-BP) database (Gene Ontology Consortium) using DAVID Bioinformatics Resources [83].

### Pseudotime inference

Monocle 2 was used to infer the pseudotemporal ordering of immune cells. We assumed the raw UMI counts were distributed according to a negative binomial distribution with fixed variance expression family to model the raw UMI count data, as recommended by the authors of Monocle 2 [83]. The Monocle 2 function BEAM was used to identify immune cells that were enriched along particular branches in the pseudotime tree. Branched heat maps were constructed using genes with q-values < 5 × 10^−5^ from the BEAM.

### Real-time quantitative polymerase chain reaction (RT-qPCR)

Eight midbrains were collected from control and RGNNV-infected fish respectively. Viral RNA was extracted from samples using the QIAamp RNA Viral kit (Qiagen, Hilden, Germany) according to the manufacturer’s instructions. cDNA was reverse transcribed with oligo(dT) and random hexamers using the PrimeScript RT Reagent Kit (Takara, Kyoto, Japan). RT-qPCR was performed using a Light Cycler②480 II (Roche, Basel, Switzerland). The RT-qPCR conditions were as follows: 95°C for 30 s, followed by 40 cycles of 95°C for 5 s, 60°C for 30 s, and 72°C for 30 s. Relative expression was determined by normalization to the housekeeping gene β-actin. We analyzed relative gene expression using the typical 2^–△△Ct^ method [84]. The primers used in RT-qPCR are listed in S9 Table.

### Fluorescent in situ hybridization (FISH) and in situ hybridization (ISH)

Sense and antisense digoxigenin (DIG)-labeled riboprobes were synthesized from the open reading frame sequence of the RGNNV CP, ptch1, robo1, kbp, plp, aplnrb, fyb1, and gatm genes using the DIG RNA Labeling Kit (Roche Diagnostics, Mannheim, Germany), and the biotin-labeled probes were synthesized from the open reading frame sequence of the slc17a7 gene using the biotin RNA Labeling Kit (Roche Diagnostics). The riboprobe sequences are provided in S1 Supplementary Sequence.

The procedures for RNA FISH were as follows: briefly, the brain tissues from grouper were fixed in buffered 4% paraformaldehyde overnight at 4°C, then rinsed twice with cold PBS, transferred to 30% sucrose solution for 48 h, and stored at 4°C. The samples dried with sucrose were frozen using dry ice and embedded in OCT compound (Sakura, USA). The tissue was sliced at -21°C, and sections were attached to cationic anti-off slides (Thermo Fisher Scientific) and then covered and incubated at 42°C for 1 day. Tissue sections were first prehybridized for 30 min, and then 250 μL of hybridization buffer containing 150 ng of DIG-labeled sense or antisense igf3 riboprobe were added to each slide. Slides were incubated in a humidified box at 42°C for 16 h. After hybridization, sections were sequentially washed twice in 2×saline-sodium citrate (SSC) (1×SSC = 0.15 M NaCl, 15 mM Na citrate) at room temperature for 15 min, then in 1×SSC and 0.1×SSC at 55°C for 1 h, and then they were mounted using Fluoroshield with DAPI (Sigma-Aldrich, St. Louis, MO, USA). Fluorescent signals from FISH were imaged using a Zeiss confocal microscope (Oberkochen, Germany).

The procedures for ISH were as follows. The slides were permeabilized three times with PBT (PBS solution containing 0.1% Tween-20) for 10 min and then treated with proteinase K in PBT (10 μg/mL) for 10 min at room temperature. They then were hybridized with sense and antisense probes at 55°C overnight. After hybridization, sections were sequentially washed twice in 2× SSC at room temperature for 15 min and then in 1× SSC and 0.1×SSC at 55°C for 1 h. Probes were detected with an alkaline phosphatase conjugated anti-DIG antibody (Roche Diagnostics), stained using the NBT/BCIP reagent (Roche Diagnostics), and imaged using the Zeiss microscope (Oberkochen, Germany).

## Supporting information

S1 Fig The distribution of basic information of each sample cell and scatter diagram of the basic information of each sample after filtration. (A) The number of genes detected in a single cell of each sample (Y axis) is distributed. (B) The total amount of UMI detected in a single cell of each sample (Y axis) was distributed. (C) The percentage of mitochondrial gene expression in a single cell of each sample (Y axis) is distributed. (D) Relationship between nUMI and nGene. The dots in different colors represent cells from different samples. X axis is the number of UMI and Y axis is the number of genes percentage. The number at the top of the figure is the Pearson correlation coefficient between the number of UMI and the number of genes/mitochondria percentage. (E) Relationship between nUMI and pMito. The dots in different colors represent cells from different samples.X axis is the number of UMI and Y axis is the percentage of mitochondria. The number at the top of the figure is the Pearson correlation coefficient between the number of UMI and the percentage of mitochondria.
(TIF)

S2 Fig Heatmap showing the cell type-specific genes are differentially expressed across the 35 clusters. Columns represent individual cells and rows represent individual genes. The expression level of a gene in different cells is represented by different colors. The more yellow the color, the higher the expression level is, while the more purple the color, the lower the expression level is.
(TIF)

S3 Fig tSNE plots showing the expression of representative marker genes are restricted to specific non-neuronal clusters among all of the cells. The expression level is color-coded.
(TIF)

S4 Fig tSNE plots showing the expression of representative marker genes are restricted to specific astrocytes clusters among all of the astrocytes subtypes cells. The expression level is color-coded.
(TIF)

S5 Fig tSNE plots showing the expression of representative marker genes are restricted to specific oligodendrocytes clusters among all of the oligodendrocytes subtypes cells. The expression level is color-coded.
(TIF)

S6 Fig tSNE plots showing the expression of representative marker genes are restricted to specific immune cells subtypes among all of the immune cells subtypes cells. The expression level is color-coded.
(TIF)

S1 Table Before and after removing cells with minimum and maximum thresholds for cells numbers, read numbers per cell (nUMI), number of genes detected per cell (nGene). (XLSX)

S2 Table Potential subtype-specific marker genes for each of the 35 clusters. (XLSX)

S3 Table Identified the genes which were significantly enriched in each of the 9 subpopulations of astrocytes. (XLSX)

S4 Table Identified the genes which were significantly enriched in each of the 6 subpopulations of oligodendrocytes. (XLSX)

S5 Table Potential subtype-specific marker genes for 4 subpopulations of immune cells. (XLSX)

S6 Table The cell numbers of 4 subpopulations of immune cells between control group and RGNNV infection group. (XLS)

S7 Table Cell numbers of 35 cluster in midbrain between control group and RGNNV infection group. (XLSX)

S8 Table The identity of marker genes used for grouper cell classification and mouse marker gene. (XLSX)

S9 Table Primers used in the riboprobes synthesis and RT-qPCR. (DOCX)

S1 Data Excel spreadsheet containing, in separate sheets, the underlying numerical data and statistical analysis for Figs 1A and 1B and 5G. (XLSX)

S1 Supplementary Sequence The sequence used for synthesis ISH and FISH riboprobes. (DOCX)
