# Supplementary figures and images for: Single-cell RNA-seq landscape midbrain cell responses to red spotted grouper nervous necrosis virus infection
Source: PLoS Pathog. 2021 Jun 29;17(6):e1009665. doi: 10.1371/journal.ppat.1009665 (PMC8241073; doi:10.1371/journal.ppat.1009665)

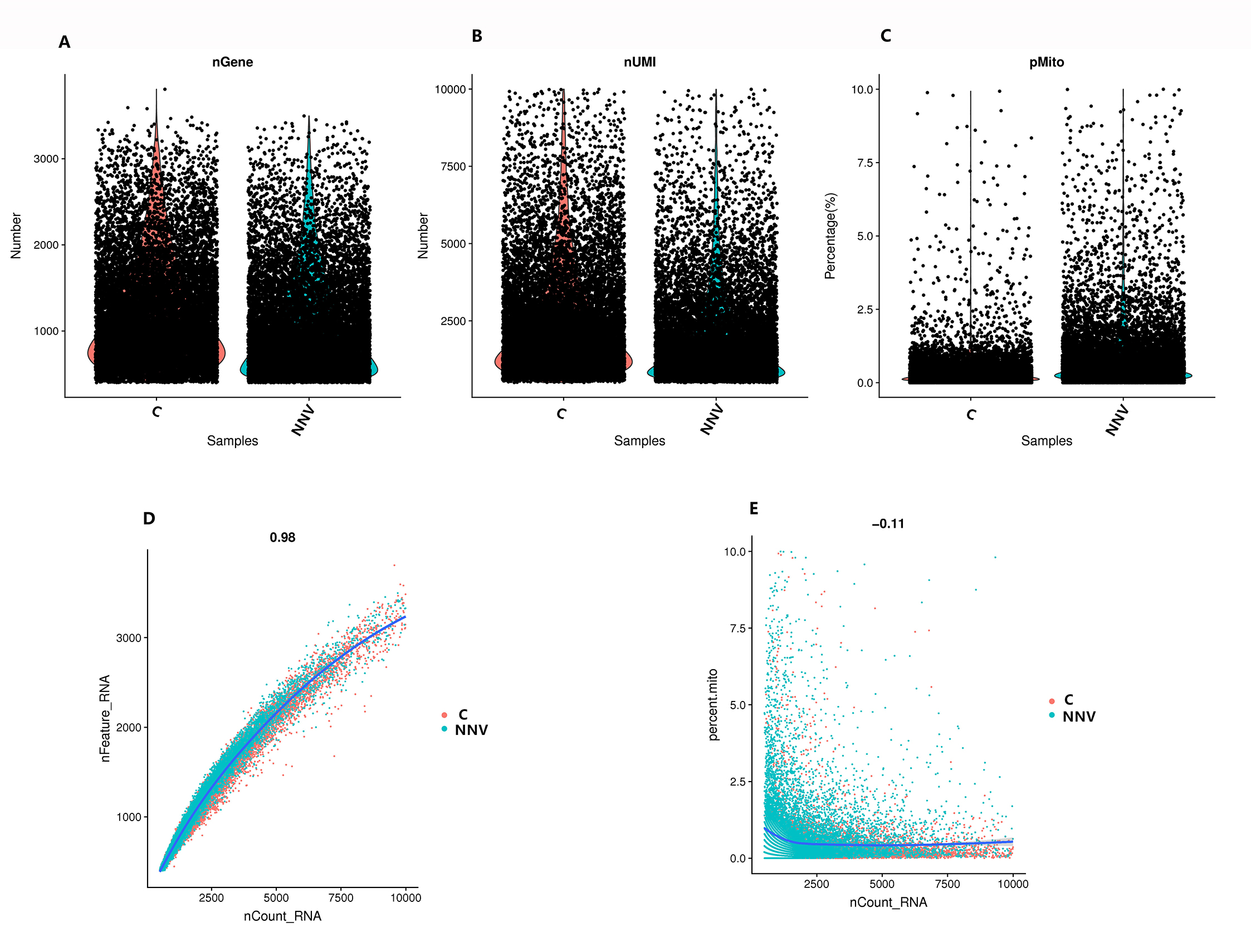

Supplement: S1 Fig — (A) The number of genes detected in a single cell of each sample (Y axis) is distributed. (B) The total amount of UMI detected in a single cell of each sample (Y axis) was distributed. (C) The percentage of mitochondrial gene expression in a single cell of each sample (Y axis) is distributed. (D) Relationship between nUMI and nGene. The dots in different colors represent cells from different samples. X axis is the number of UMI and Y axis is the number of genes percentage. The number at the top of the figure is the Pearson correlation coefficient between the number of UMI and the number of genes/mitochondria percentage. (E) Relationship between nUMI and pMito. The dots in different colors represent cells from different samples.X axis is the number of UMI and Y axis is the percentage of mitochondria. The number at the top of the figure is the Pearson correlation coefficient between the number of UMI and the percentage of mitochondria. (TIF) [file ppat.1009665.s001.tif]

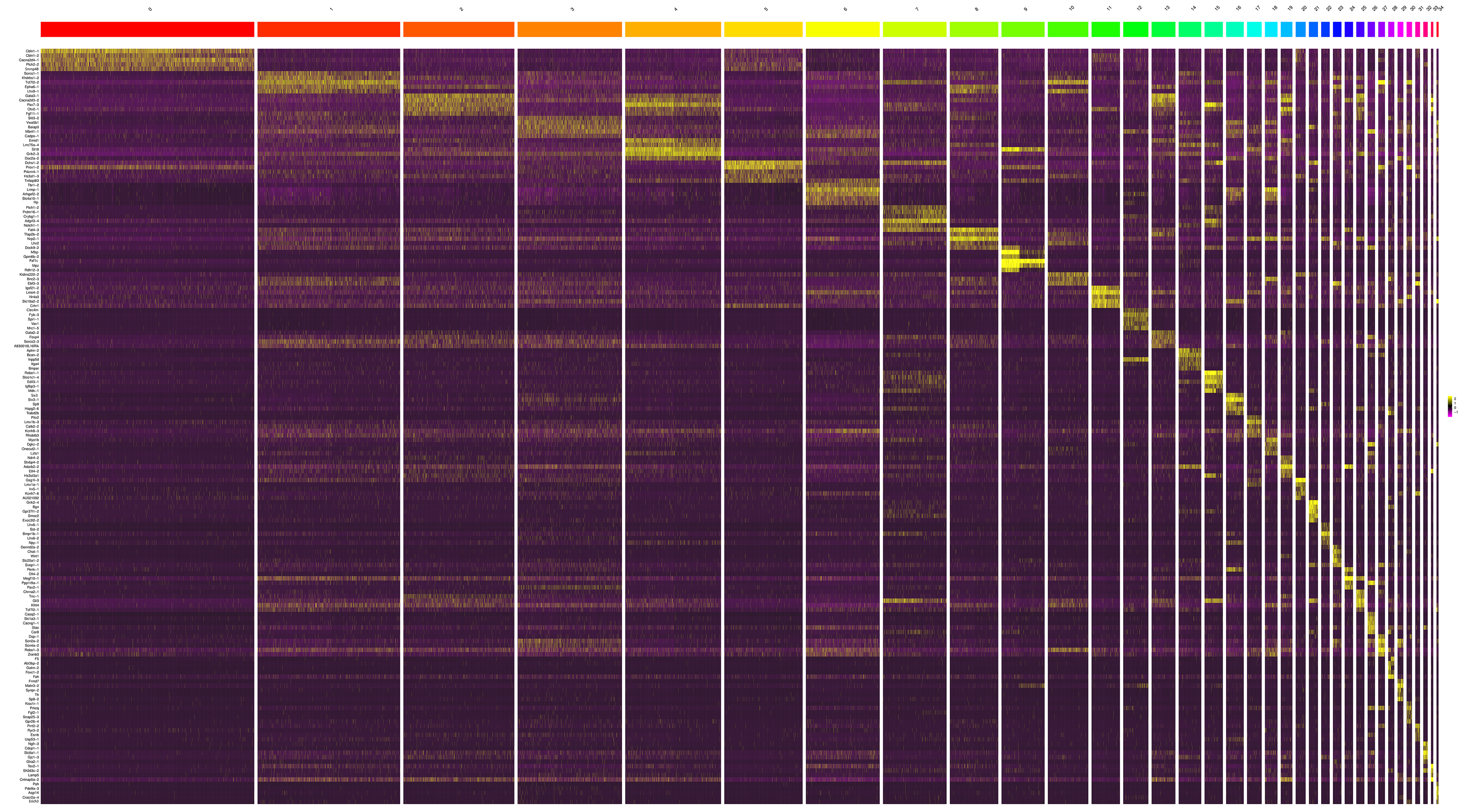

Supplement: S2 Fig — Columns represent individual cells and rows represent individual genes. The expression level of a gene in different cells is represented by different colors. The more yellow the color, the higher the expression level is, while the more purple the color, the lower the expression level is. (TIF) [file ppat.1009665.s002.tif]

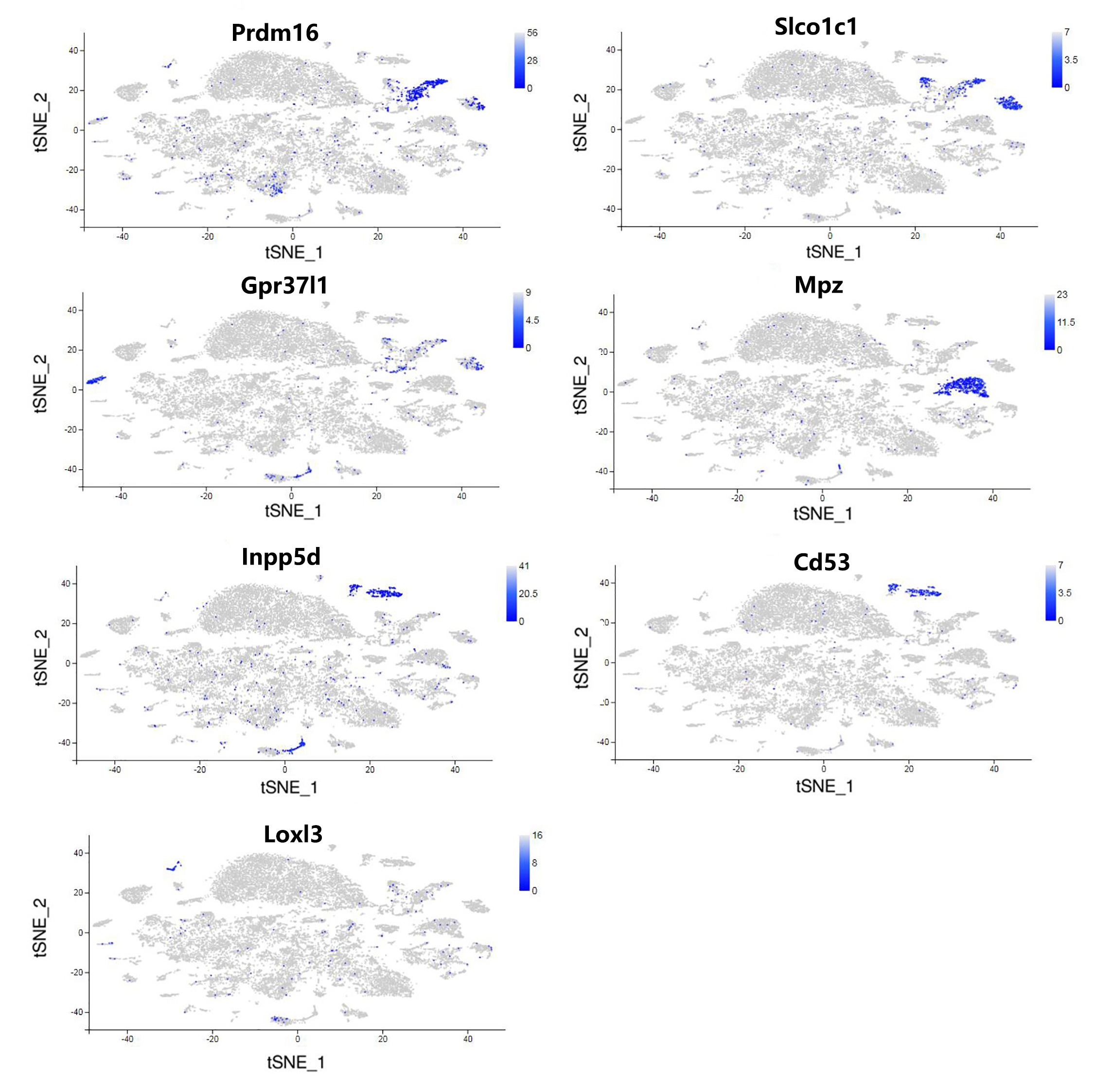

Supplement: S3 Fig — The expression level is color-coded. (TIF) [file ppat.1009665.s003.tif]

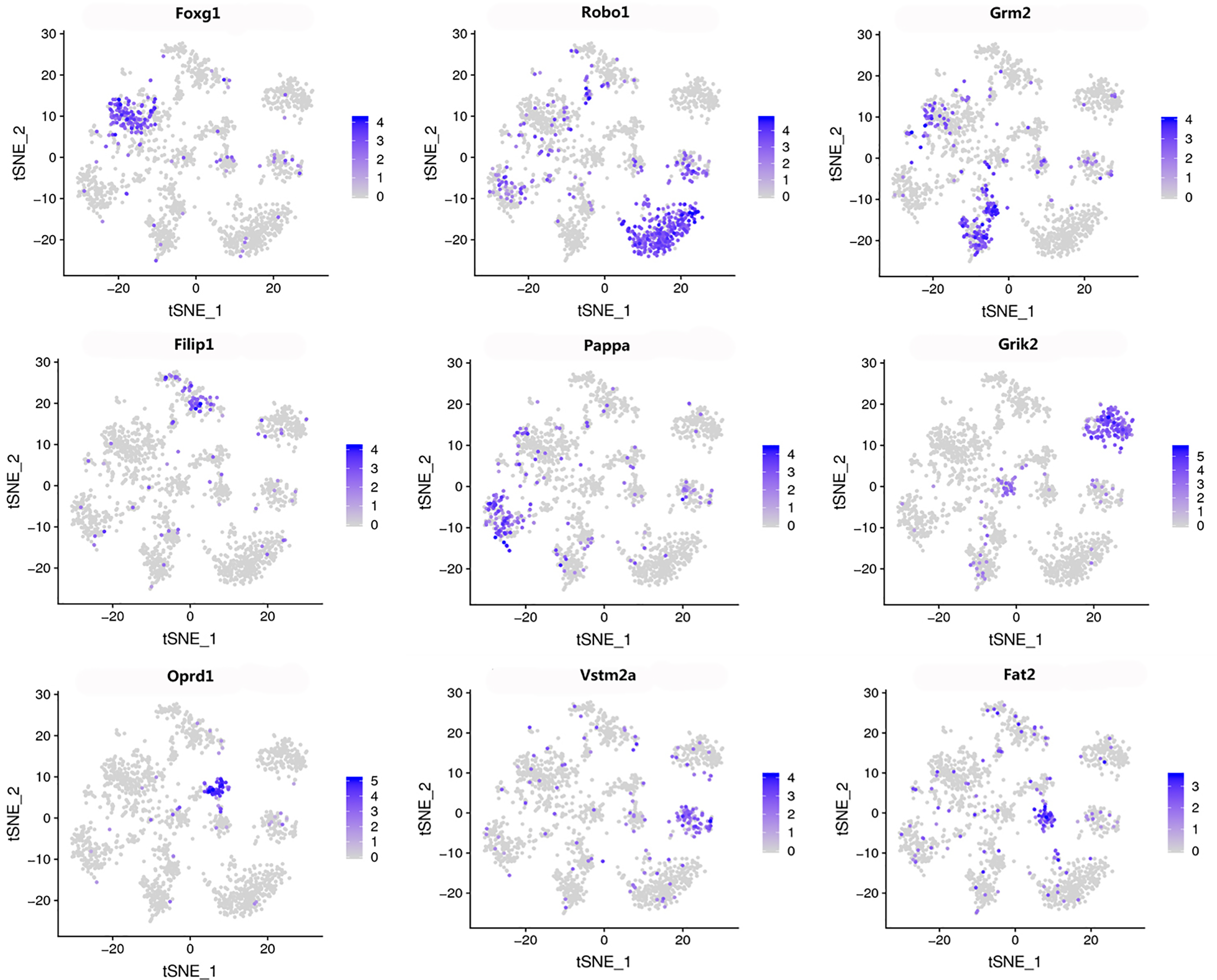

Supplement: S4 Fig — The expression level is color-coded. (TIF) [file ppat.1009665.s004.tif]

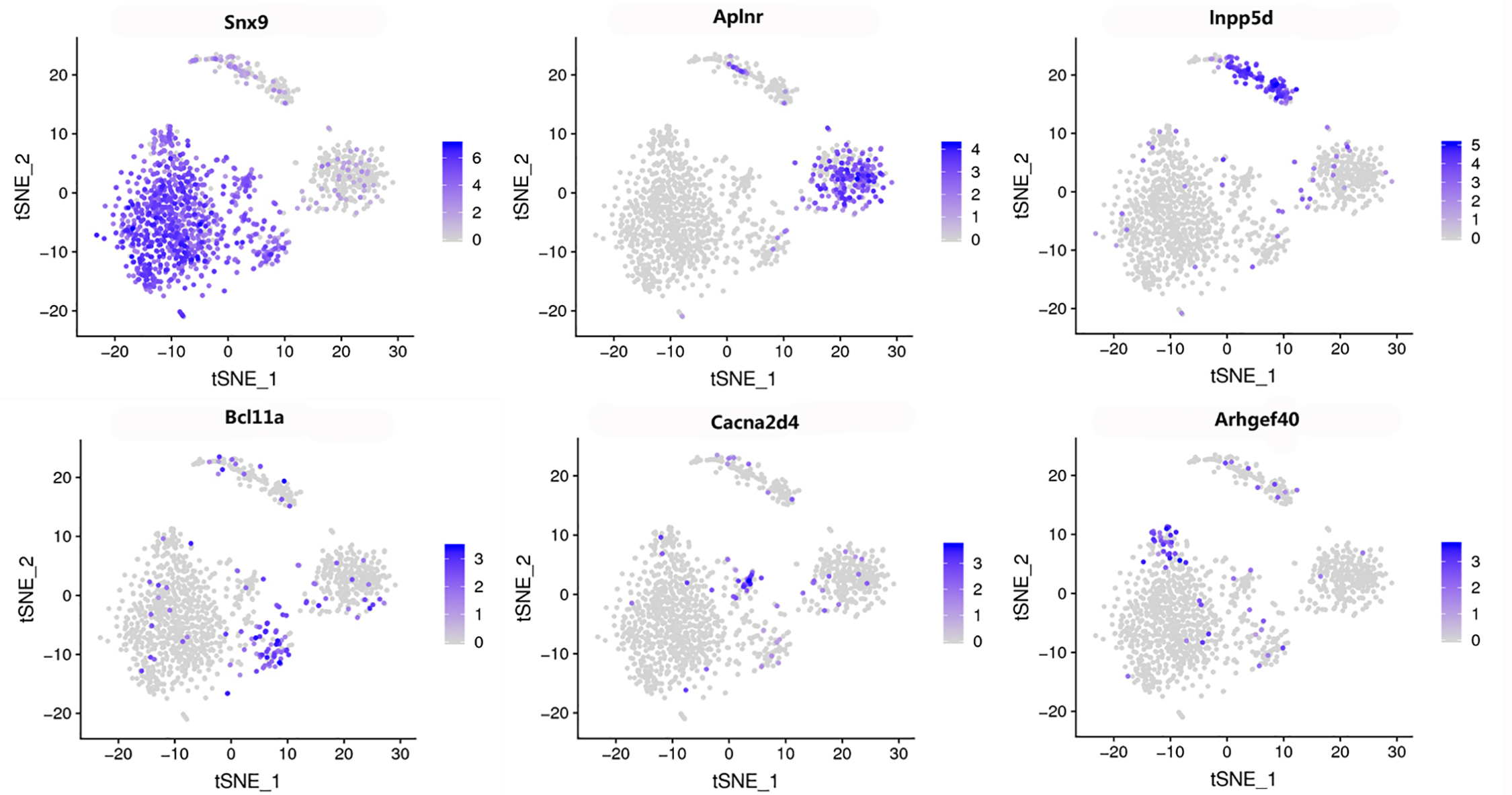

Supplement: S5 Fig — The expression level is color-coded. (TIF) [file ppat.1009665.s005.tif]

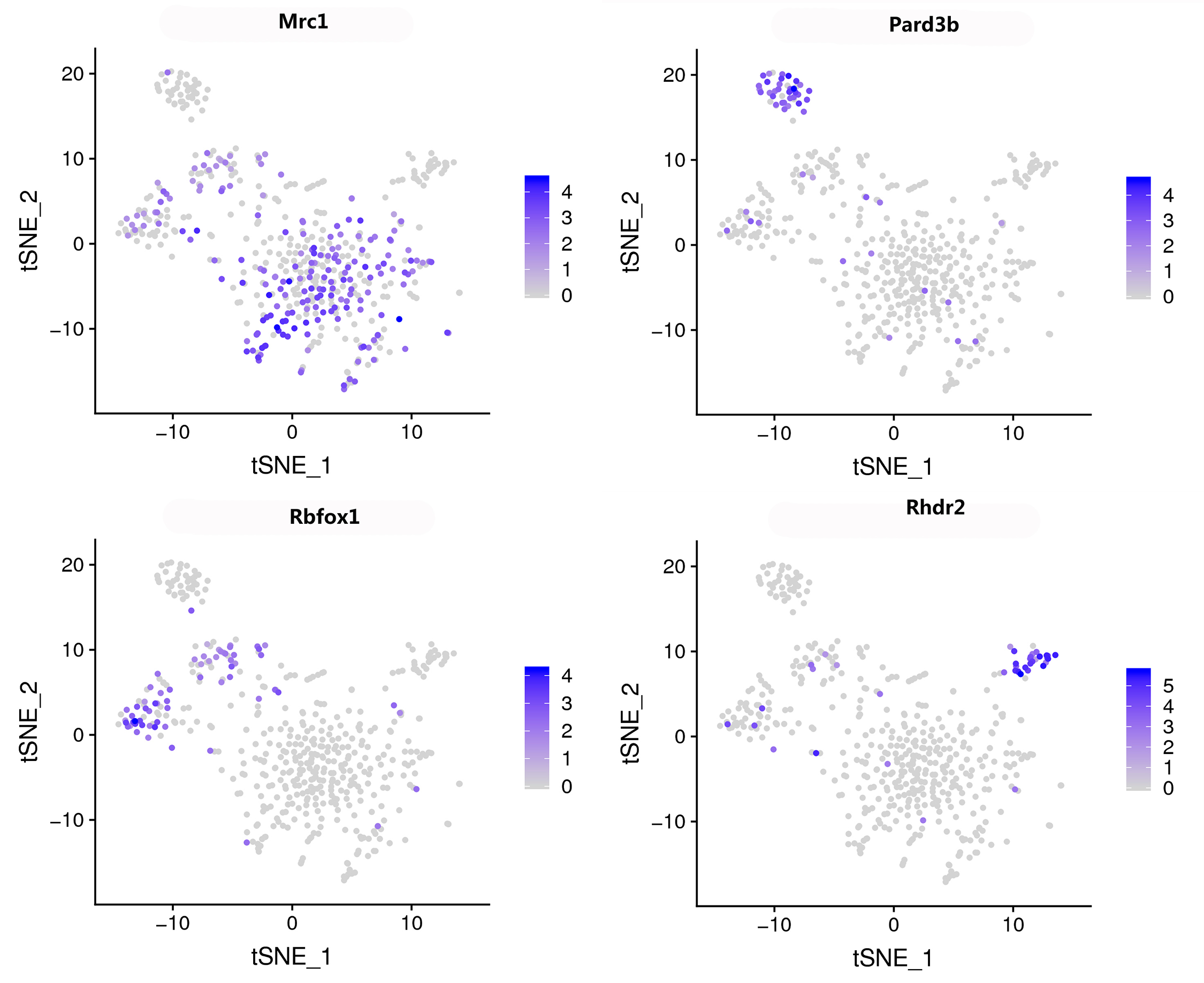

Supplement: S6 Fig — The expression level is color-coded. (TIF) [file ppat.1009665.s006.tif]
