# Supplementary material for: Single-cell RNA-seq landscape midbrain cell responses to red spotted grouper nervous necrosis virus infection
Source: PLoS Pathog. 2021 Jun 29;17(6):e1009665. doi: 10.1371/journal.ppat.1009665 (PMC8241073; doi:10.1371/journal.ppat.1009665)
Supplement: S9 Table — (DOCX) [file ppat.1009665.s015.docx]

S9 Table. Primers used in this study

| **The primers used in the riboprobes synthesis** | |
| --- | --- |
| Rib-CP-F：CAGAACAGTCCGACCTCAGTAC | Rib-CP-R：GTCAACCCTAGTGCAGACAGTG |
| Rib- Ptch1-F：GACTCTGAGTATTACTCTGAG | Rib- Ptch1-R：CTCCAAGTCCTGGAGCTCTAG |
| Rib- Robo1-F：GAAGGCTCCAGTCGTTCTAC | Rib- Robo1-R：GGTGTTTCTGCCAGGATAGTG |
| Rib- kbp -F：TACCATTGTCCTCCTGGCTTG | Rib- kbp -R：GAGGTTTGTTTGCATCGCTG |
| Rib- Plp -F：CTTAGGTTGTTATGATTGCTG | Rib- Plp -R：GCAAGACTGCGTAGTTGTAGG |
| Rib- Aplnrb -F：CTGTGCAAGATCAGCAGCTACG | Rib- Aplnrb -R：AGAGACTGAGCCTCTGTCTTC |
| Rib- Fyb1-F：GGACATTGAGGATAATGAGGAC | Rib- Fyb1-R：CATCATAGTCATCATCTTCTTG |
| Rib-Gatm-F：AATGAGATTATCGAGGCTCC | Rib-Gatm-R：GGTTGTCCAGCAGTGAAAGC |
| Rib- Slc17a7-F：CGTCAGGAAGCTCATGAACTG | Rib- Slc17a7-R：CACATACTCCTCAGACTTATCC |
| Rib- Mrc1-F：ATAGGGACACATATTTTTGTTCC | Rib- Mrc1-R：GCAGATAAAGTTGAGTTTGTCC |
| **The primers used in the RT-qPCR** | |
| q-slc6a6-F: GATCGAGGACATAGGACCT | q-slc6a6-R: GTGTTCCTGCATCAATCCAC |
| q-ptprf -F: GGAGCTTTGACCACTATGCA | q-ptprf -R: CAGCTGCGTCTTCTCCAGCTT |
| q-arhgap6-F: GGAACGCAGCACATGTATGA | q-arhgap6-R: AGAGTCCATGCAGCTACACA |
| q-vipr2-F: GTGGTGAGGATCCTGTCTAC | q-vipr2-R: AGTTGAAGAACACCAGCGTG |
| q-hsp90-F: GCTGTTCAAGTTCATGGTGAC | q-hsp90-R: ACAGACAAGCCTGGACTCAG |
| q-kdm6b -F: GACACACCATCCTTGGCATG | q-kdm6b -R: GATCCACACCAAGTCTCCAG |
| q-cbx7-F: CTGTGCCACACATGCACAAAG | q-cbx7-R：GGCTAGTGAAAAGAGTGGAC |
| q-TNFa-F: CCTGGTGATGTGGAGATG | q-TNFa-R: GTCCGACTTGATTAGTGCTT |
| q-TLR18-F: CTGAAAGAGCTCTACATGTCC | q-TLR18-R: CATCTGCTTTGTCTGGATGACC |
| q-LITAF-F: TGTTGCCTTTGCTCCTGCAC | q-LITAF-R: CGTAGATGACTCTGTTGCAG |
| q-NFκB-F: AACCTCACCGAGCCCATTA | q-NFκB-R: TTGTCACTCAGCCTGTATTCATCT |
| q-IL1R-F: GACGTCATTGCTGCTAGAGT | q-IL1R-R: CACCAGAATCACTTGAGGTTC |
| q-CD97-F: CTGCCTGCATCATCATCAT | q-CD97-R: CTGCCAAAGATGGTGAACAGG |
| q-CP-F: CAACTGACAACGATCACACCTTC | q-CP-R: CAATCGAACACTCCAGCGACA |
| q-RdRp-F: GTGTCCGGAGAGGTTAAGGATG | q-RdRp-F: CTTGAATTGATCAACGGTGAACA |
| q-β-actin-F: ACCATCGGCAATGAGAGGTT | q-β-actin-R: ACATCTGCTGGAAGGTGGAC |
