# Supplementary material for: Single-cell RNA-seq landscape midbrain cell responses to red spotted grouper nervous necrosis virus infection
Source: PLoS Pathog. 2021 Jun 29;17(6):e1009665. doi: 10.1371/journal.ppat.1009665 (PMC8241073; doi:10.1371/journal.ppat.1009665)
Supplement: S1 Supplementary Sequence — (DOCX) [file ppat.1009665.s017.docx]

**Riboprobe sequence of RGNNV:**

CAGAACAGTCCGACCTCAGTACACCCGCACGCTCCTCTGGACCTCGTCGGGAAAGGAGCAGCGTCTCACGTCACCTGGTCGGCTGATACTCCTGTGTGTCGGCAACAACACTGATGTGGTCAACGTGTCAGTGCTGTGTCGCTGGAGTGTTCGACTGAGCGTTCCATCTCTTGAGACACCTGAAGAGACCACCGCTCCCATCATGACACAAGGTTCCCTGTACAACGATTCCCTTTCCACAAATGACTTCAAGTCCATCCTCCTAGGATCCACACCACTGGACATTGCCCCTGATGGAGCAGTCTTCCAGCTGGACCGTCCGCTGTCCATTGACTACAGCCTTGGAACTGGAGATGTTGACCGTGCTGTTTATTGGCACCTCAAGAAGTTTGCTGGAAATGCTGGCACACCTGCAGGCTGGTTTCGCTGGGGCATCTGGGACAACTTCAACAAGACGTTCGCAGATGGCGTTGCCTACTACTCTGATGAGCAGCCTCGTCAAATCCTGCTGCCTGTTGGCACTGTCTGCACTAGGGTTGAC

**Riboprobe sequence of** **Ptch1:**

GACTCTGAGTATTACTCTGAGATGACCACCACATCAGGGATCGGGGAGGAGGACTATAAGTACTGTGACCGGAGCGCCTACATAGCATCGCACAACAGCGCTCCACCTGCAACATCTCACATACTGCTGGAAGCCAGCAAGAACCCCAGCTTCCCCAAGCTAACGGTGGTGAAGCCGTTCAGAGAAAACGCAACAAGCGGCGGTGGAAGGATAGAACCGTTAAATGAACCTTCCCACAATGCACATTCATCTCTCGGCTCCCAGGTTACATGTTGGGATGGAAACAAGCGGGAGCAGCAGCCGGGCCTCCAGAGGCTGCAGGCGCAACCCTTACCCAGCGACAAACCTCACTTCCCTGGGAGGACTTGTCAAAGCGGCCCCAGGCTGCAGAACGGCAGAGGGCCCCAGCCAAACAGGACTAAAGGCCCGAGCTATAGCAGT

AGCAACTCCACCCTGCCGATGCAACAAGGCACTGCGGCGGGGCCCGTTACCATGGTGACGGCCACCGCCTCCGTGACGGTGGCTGTGCATCCGACCTTGCCGGGGGCGGCATACCAAGGCTACATGCACGAAGGTTTTGACACGGACAGCGAGTCAGACTGTTTTGAAGCTGCTAAGAGGACTTGTGGTGACAAAACAAACTTTTCTTCATGTAAGAGAGACTCTCTAGAGCTCCAGGACTTGGAG

**Riboprobe sequence of** **Robo1:**

GAAGGCTCCAGTCGTTCTACAAAGTTAACAGTGGACATGGGCTCTCTGCAGTCAGTGTGTGCTGCGGCAGGCCACCGTGGGAAACCAGGACCCTCCACTAACAACAGCAGTTTCCCTTCCTACAACCACCTGTCTACATCATATTCAATGGATGACGAACAGGGTGGCACATTGACAGCACAGGAAGCCACCCAGTATCTGGAGCTCAGCCCTAAACCTGAGAGATGCAGCGCCCTGCCTCAGCAGCATCCGTCCCTGCCAAACCCGTTCTCCTCCACGCTGGGCTACATCTCTGGGACTGGCCGCTCCACTCAGCTGGAGGATGACTCCACCACCGATGACCCTGAGGTGCCACCAATCGGCTTGCGACGTGCCCGCCTCCAGAGCACACCTTCCTCCTGCTACAGCGAATGGGACAGCTCACTGTGGAACACCTGGAGCTCAGCAATGGACAGCAACATGGCCAGCGCTCGCACCAGCCTCATCAGCTCAGTGGACAGCTGCTATACTAACGACAGCGCCAACTTTGCCCGCTTGCTGGCTGCGGCAGCAGATACCATGAGTGGGGCCTCTTTGTCAGACTTCTCTCCACCGGCCTCCCCCCTCAGCGCCTTGTATCCGTCATTTCGTGCAGAGGGCGACTCGTACGGCGAGCTGGAGCCTGTTCCTGCGTGGGACTGGAGCATGGCGTGGGTGGAAGAAATGGAGGCTCAGTACAGAGCTCACTATCCTGGCAGAAACACC

**Riboprobe sequence of** **kbp:**

TACCATTGTCCTCCTGGCTTGTTATTTCTCCAACCTCAACTCCTCTAAGACCTCTGAGTCCACTCACCTGACAGTGAAAGGGTTTGAGGACTTGGCCAATCAGGACATGATTGAGTACGGCTGTTTGGCCGGCTCCTCCCCTCTTTCCTTCTTCAAGAATTCAAACAACCCAGTGTATCGCAGAATCTATGAGCACATGGAGAGAACTAAGAGTTTTGTGTCATCTATGGACGAAGGGGTCAGACGTGCAAAAGAGGGAAACTTTGCCTTCATCGGAGAGTCTGTTTCTTTGGACCTGGCAGTAGCACGTTATTGTGAGCTAGTCAGAGCACATGAAGTCGTTGGAATGAGGGGATACAGCATCGCTGCCCCCCTTGGCTCACCCATCATAAAGAACCTCAGTGTGGCCATCCTTCAGCTGAGTGAGGCGGGGGAGCTGGCTTACCTGCGAAGCAAATGGTGGGCCAGCAGCTGCCTAGCAAACAGGGCCAAGTCTTCAGCTGTGCAGCCACACAGCCTCAAAGGGATGTTTCTGGTTCTTTCCCTGGGCCTGGGGCTGGGTGCACTGCTGGCTGTCCTGGAGCTCACCTCCAAGAGCCGCAGAAGTGCAGCTGAGCAGAAGAAATCCTGCTGCTCTGTGCTGACTGAAGAACTGAGTCTGCGCTTGAGGACCAGCGATGCAAACAAACCTC

**Riboprobe sequence of** **Plp:**

CTTAGGTTGTTATGATTGCTGTATCCGGTGCATTGGGGCAGTGCCCTACCCATCCCTGGTGGCCACCCTGCTGTGCTATGCTGGCATGGCATTATTTTGTGGCTGTGGGCATGAAGCGTTGTCCCAGACCGAAGTCCTCGTCGAGACTTACTTCGCCCGCAACGTTCAAGACTTTGTGGTCATGGCCTCCTTTATCAAATACTTCCAGTACGTGATCTATGGCCTGGCATCATTTTTCTTCCTCTATGGTATCCTGCTGCTGGCTGAGGGTTTCTACACCACGAGCGCTGTGAAGCAGACCTTTGGTGAATTCAGGAGCACCCAATGTGGCCGCTGCCTCAGCCTGACGTTCATCATAGTGACGTACATCTTGGCCTTCATCTGGCTGGCGGTGTTTGCCTTCACTGCTATCCCAGTCTTCTTCTTGTTCAACATGGAGCAGACCTGCCACAACATCAACATCCTGGCTGAAACAACCCCCAGCATTAATCAACACGGCTGGATTTGCATGGACGCCAGGCAGTATGGTCTGCTTCCTTGGAATGCAATGCCAGGCAAGGCTTGTGGAATGACCTTGGCATCTATTTGCAAAACCAGCGAATTCTACGTCACCTACGACCTGTACATAGCTGCATTCGCTGGAGCAGGGGTCACTCTCTTAGCACTGTTTCTGTATCTGGCTGCGACTACCTACAACTACGCAGTCTTGC

**Riboprobe sequence of** **Aplnrb:**

CTGTGCAAGATCAGCAGCTACGTGGTCCTGCTCAACATGTACGCCAGCGTCTTCTGCCTCACCTGCATGAGCTTCGACCGCTACCTGGCCATCGTGCACTCCTTGTCCAGCACCCAGCTGCGCACCCGTGGCCACATGCAAGCCTCCATAACAGCCATCTGGATGCTGTCCGGTATCCTGGCTGCCCCGACTCTGCTGTTCCGTACAACCAAAAATGAACCAACCAGCAACCGCACATCCTGCGCCATGGACTTCAGCCTGGTGATGACCAGGAAAGATCAAGAGAACCTGTGGATCGCAGGTCTCAGCATCTCCTCTTCAGCTCTGGGCTTCCTTCTACCTTTCTTGGCGATGATGGTGTGCTACGCCGCCCCCCCCCCCCCCCGCCACTTCAACACCCTGCGCAAAGAGGACCAGCGTAAGAGGAGGCTGCTGAAGATCATCACCACGCTGGTAGTGGTGTTCGCTGTCTGCTGGATGCCCTTCCATGTTGTGAAGAGCGCCGACGCTCTCTCCTACCTGGAGCTGTTTCCTGCGACCTGTGCCTTCCTGCGCTTCCTGCTGCTGGCTCACCCCTACGCCACCTGCCTTGCCTACGTCAACAGCTGCCTCAACCCCTTTCTTTACGCCTTCTTCGACCTGCGCTTCAGATCCCAGTGTCTGTGCCTGCTCAACCTGAAGAAGTCCCTGCACGCGAGCCCTATCAGCTCCCTGTCCTCTCAGAAGACAGAGGCTCAGTCTCT

**Riboprobe sequence of** **Fyb1:**

GGACATTGAGGATAATGAGGACTTCTATGATGACATTGATAGGAACGAGTCCTGT

AGCGACAACGGTTCACACTGTATGGATGGGGAAGATGATGAAGTGTATGAGTTTATTGAC

GAGGACCAGGTGGAGCAAAATCAATTACATGCTGGGAAGCAAAACAAAAAAGATGCAAAG

AGGCAGCGGGAGCAGGAGAAGAAAGAGCAGATGGAGCGTCAGAAAAAAGAAAATGAGTTG

AGGAAGAAATTTCAGTTGCAAGGGGAGGTGGAGGTTATTCATACAGCCAAAGTCCGGCAT

GACTGGTATGGAGGAGGAAAACTGGACCTCAGCGTACGACAAGGAGAGAGCGTGGAGATC

CTCAGAGTGAAGAATAACCCCGGAGGCAAATGGTTGGCTCGCTCTCTGAACGGAAACTAT

GGATACATCAGTAACACATGTGTGGATGTTGACTATGAAGCAGTGAAGCGCAAAGTGCTC

CAGTCCAGGAAAATAGACACATCAACATTGCCTCCACCACCTCCAGACCCCCCAATGATG

TTGAATATAGAGTTGAACAGTAGCAACAGCATGCTTCAAGAAGATGATGACTATGATG

**Riboprobe sequence of** **Gatm:**

AATGAGATTATCGAGGCTCCTATGGCCTGGAGGGCTCGCTTCTTTGAGTACCGAGCCTACAGACCTCTGATCAAGGAGTACTTCAGAAAAGGTGCTAAGTGGACCACTGCTCCCAAACCCACTATGGCTGATGATCTGTATGATCAGGACTACCCCATCCGCACAGTGGAGGACAGACACAAGCTGGCTGCCGAGGGGAAGTTCGTGACTACAGAGCACGAGCCCTGCTTTGATGCTGCGGACTTCATCCGAGCTGGGACGGACCTTTTCGTCCAGAGGAGTCAAGTTACAAATTACATGGGAATTGAATGGATGCGCCGCCATCTGGCTCCAGACTACAAGATCCACATCATTTCATTCAAGGATCCTAACCCCATGCACATTGATGCCACGTTCAACATCATCGGGCCGGGACTGGTGCTGTCAAACCCTGATCGTCCATGTCGCCAGATTGACATGTTCAGGAAGGCTGGCTGGACTGTTGTTAAACCTCCGACGCCTCTGATTCCTGATGACCACCCCCTGTGGATGTCCTCCAAATGGCTGTCCATGAACGTCCTGATGTTGGGTGAGAAGCGTGTTATGGTTGACGCCAATGAAAGCACCATCCAAAAAATGTTTCAGAGCCTCGGTATCGAGACCATAAAGGTGAACATTCGCCATGCCAACTCCCTGGGTGGTGGCTTTCACTGCTGGACAACC

**Riboprobe sequence of** **Slc17a7:**

CGTCAGGAAGCTCATGAACTGTGGAGGTTTTGGGATGGAGGCCACCCTCCTGCTGGTAGTGGGATACTCTCATTCAAAAGGTGTTGCCATTTCCTTTTTGGTCCTCGCTGTGGGTTTCAGTGGATTTGCCATCTCAGGGTTTAATGTCAATCACTTGGATATCGCCCCTCGATATGCCAGCATACTGATGGGCATCTCAAACGGGGTGGGAACACTATCTGGAATGGTGTGTCCTCTCATAGTGGGAGCCATGACCAAACACAAGACACGTGAAGAGTGGCAGTATGTCTTCCTTATAGCTTCCCTCGTTCATTATGGAGGAGTGGTTTTCTATGGACTCTTTGCATCGGGAGAAAAGCAAGCATGGGCGGACATAGAGGACACGAGTGAGGAGAAGTGTGGTATTATAGATGAGGATGAACTGGCCAATGAAACAGAGGAGCTCTACCGTGGAGGTGGGCAATACGGGGCCATAAACCAACCAGTTGTTGGATCCAACGGAGGAGGGGCAGGAGGAGGAGGAGCTGGTTCTGGATGGGTGTCGGACTGGGATAAGTCTGAGGAGTATGTG

**Riboprobe sequence of MRC1:**

ATAGGGACACATATTTTTGTTCCTATAAGAATGCCGTGGCTTGATGCTCAGACTTACTGTAGAAAAAATTACACCGACCTTTCTTTTGCTGATAGTCAGAGTGATCAAGACAGGCTCGTGGCGGCTGCAGGTGTAAACATTACAGACGGATGGATTGGTCTCCATCGAGATCCCAGCAACATTAACAGCTGGAAGTGGTCAGGAGGGGGCTACGTTACATACAATAACTGGGGAGGAGGGCAACCAGATAATGCGAATGATAATGAGCCTGTTGGACATATCATGTCTGATGGAAGATGGAATGATATCAGAGAAACCAAACACAAGCCTTTTTACTGCATCAGTATTAAGGCAGTGGAGGTGAGGATGACGTGGGAGGACGCTCTGGAGCACTGCAGAGAAAGCCACACTTACCTCACCAGCCTACTCTCTGAAAACGAGTGCCTTCTGGCCCAGAGAGAGATCCAGAAAGCTCACATCACCAAGTGGGTGTGGATCGGCCTTCGTTACCTGGAGGACCGCTGGTTTTGGGTGAACAATAACCCTCTGGTGTACCAGGCCTGGTCCAAAGATGGAGCTCAGGACCGCCAGTGTCCAAAACAGAGACGCTGTGGAGCTTTAACCAAAGATGGACTGTGGGAGAACTTGGACTGTCAGGACAAACTCAACTTTATCTGC
